# Reconstructing the glioblastoma microenvironment in heterotypic 3D spheroids: a multicellular model to study tumor-stromal crosstalk

**DOI:** 10.3389/fbioe.2026.1852454

**Published:** 2026-07-03

**Authors:** Yuliya Nikitina, Alina Kazakova, Anastasia Leonteva, David Sergeevichev, Maria Bogachek, Sergey Vladimirov, Natalia Vasileva, Vladimir Richter, Anna Nushtaeva

**Affiliations:** 1 Scientific Center of Genetics and Life Sciences, Sirius University of Science and Technology, Sirius, Russia; 2 Laboratory of Biotechnology, Institute of Chemical Biology and Fundamental Medicine, Siberian Branch of the Russian Academy of Sciences, Novosibirsk, Russia; 3 Meshalkin National Medical Research Center, Novosibirsk, Russia

**Keywords:** 3D-model, cytokines, GBM, glioma, heterotypic, SDF1, spheroid, tumor microenvironment

## Abstract

**Background:**

The complex interplay between tumor cells and the stromal components of the glioma microenvironment necessitates the development of sophisticated *in vitro* models capable of modelling key aspects of cellular interactions that occur beyond the limitations of conventional monocultures.

**Methodology:**

The development and characterization of homo- and heterotypic 3D spheroid models incorporating CCF-STTG1 astrocytes, HMC3 microglia, and U87MG glioma cells was undertaken. The assessment of morphological, molecular, and functional properties was performed via flow cytometry, cytokine arrays, ECM analysis and invasion assays (Matrigel™/gelatin).

**Results:**

Heterotypic spheroids have been observed to spontaneously self-assemble into a spatially polarized architecture, with microglia and glioma cells segregating into distinct compartments, a pattern suggestive of the cellular topology at the invasive front. The morphological, molecular, and functional properties of the generated 3D models recapitulated several established features associated with *in vivo* tumors, including growth, invasion, resistance to chemotherapy, and metabolic reprogramming alongside the expression of stemness markers, and key pro-invasive mediators (MMPs, SDF-1α, VEGF). Secretome profiling revealed a marked, non-additive upregulation of chemokines (IP-10, MIP-1α) and the emergence of novel correlations (HGF/SDF-1α, MCP-1/LIF), indicating potential modulation of paracrine networks involved in immune cell trafficking in the heterotypic setting. The initial formation of a rigid ECM matrix appears to be initiated by microglia, while the supply of fibronectin and laminin may be linked to astrocytes exhibiting some features of reactive gliosis, which could help organize invasion pathways.

**Conclusion:**

These heterotypic 3D spheroid models offer a stroma-enriched, reproducible platform for the analysis of stromal contributions to glioma progression and for exploratory preclinical evaluation of therapeutic strategies.

## 1 Introduction

Glioblastoma (grade 4 glioma, according to the WHO) classification of central nervous system tumors) is the most common malignant tumor of the central nervous system. It is characterized by rapid proliferation and aggressive growth (Louis et al., 2021). Treatment options are limited, and the prognosis is poor, which is largely attributed to its microenvironment (Sharma et al., 2023). The glioma microenvironment is characterized by “immune exclusion,” a state marked by the presence of low levels of infiltrating immune cells. This condition may contribute to the limited efficacy of various therapeutic approaches (Sampson et al., 2020).

The tumor microenvironment (TME) is a heterogeneous and complex system with cross-interacting components that contribute to tumor progression and invasion. The TME of glioma comprises the following components: the immune system comprises various cell types, including tumor-associated macrophages and microglia (Joseph et al., 2021; Salmeron-Moreno et al., 2026; Szklarczyk et al., 2025), neutrophils, myeloid suppressor, T-, and natural killer (NK) cells. In addition, there are non-immune cells such as endothelial, glioma-associated mesenchymal stem cells, cancer-associated fibroblasts, pericytes, neurons, and astrocytes. The third component is the non-cellular element, which includes extracellular matrix proteins (Lootens et al., 2024).

Recent studies have demonstrated that microglia and astrocytes play a pivotal role in the proliferation of glioma tumor mass (Matias et al., 2018). Microglia, a type of immune cell present in the TME, have been shown to play a role in promoting tumor growth and invasiveness by secreting cytokines and growth factors, including IL-6, TGF-β, and VEGF. Tumor-associated microglia have been observed to protect cancer cells from radiation-induced apoptosis by secreting immunosuppressive cytokines, including IL-6 and TNF-β. This phenomenon contributes to the development of resistance to radiation and chemotherapy (Wang et al., 2024). Astrocytes, a type of glial cell in the central nervous system, have been shown to play a significant role in the TME by promoting angiogenesis. Astrocytes have been shown to produce immunomodulatory substances, including TGF-β and IL-10, which have been found to inhibit the activity of immune cells involved in antitumor immunity, such as T cells and NK cells (Matias et al., 2018). A comprehensive understanding of the cross-talk between tumor cells and astrocytes is imperative for elucidating the mechanisms underlying glioma immune evasion. This understanding should be a fundamental consideration when formulating novel immunotherapy strategies. Consequently, the development of more effective anticancer drugs necessitates the consideration of not only tumor cells but also microenvironmental cells, thereby creating more physiologically relevant conditions *in vivo*.

A critical challenge in the development and implementation of effective anticancer therapeutic approaches is the lack of adequate *in vitro* model systems. Recent progress in the development of new effective treatments is accelerating; however, the number of testing platforms capable of accurately mimicking the tumor and its microenvironment remains limited (Rodrigues et al., 2024). In the context of anticancer drug testing, a significant number of experiments continue to be conducted using two-dimensional (2D) cultures, xenografts, or syngeneic animal models (Pinto et al., 2020). Nevertheless, 2D models are incapable of replicating the intricacy of tumor structure and the interaction between tumor cells and the immune system. It has been demonstrated that animal models do not always adequately reflect human-specific events, thereby limiting their applicability (Rodrigues et al., 2024). Researchers have developed new genetically engineered and humanized models for targeted studies of the tumor microenvironment alongside traditional *in vivo* rodent models (e.g., intracranial xenografts) (Sharma et al., 2023). Moreover, the utilization of experimental animal models can incur substantial financial costs and present considerable operational challenges. Their application gives rise to ethical considerations pertaining to animal welfare, especially within the framework of the 3Rs (replacement, reduction, refinement). Furthermore, improvements to existing three-dimensional (3D) cellular tumor models have also helped to bridge the gap between fundamental discoveries and their application to therapeutic modelling of glioma (Vakhshiteh et al., 2023). Heterotypic 3D tumor spheroids, consisting of multiple cell types, have become a more popular and sophisticated model for mimicking tumor heterogeneity and physiology *in vivo* than homotypic spheroids (composed of a single cell type) (Leonteva et al., 2025; Garcia-Saez et al., 2026; Leonteva et al., 2026). In contrast to animal models devoid of an immune system (xenografts) or the microenvironment of human cells (syngeneic models), *in vitro* 3D models permit the effective utilization of both immune cells and human cells (Heinrich et al., 2024). Furthermore, they facilitate a more profound examination of cellular interactions, resistance mechanisms and other biological characteristics that are not possible to investigate with *in vivo* studies.

The 3D models created must accurately reflect the spatial structure of the tumor and possess the functional characteristics of a malignant tumor, including the ability to grow, invade, and secrete chemokines that support tumor development and its metastatic potential. A number of 3D heterotypic models have been developed previously (Heinrich et al., 2024; Diep et al., 2023). For example, a recent study created a heterotypic model from glioma and microglia cells (Garcia-Saez et al., 2026). The present study corroborated the model’s substantial pertinence to the functional characteristics of the tumor *in vivo*, encompassing the impact of microglial cells on proliferation and invasion. However, this model does not include a critical component of the TME: astrocytes. In their reactive state, these cells significantly influence the aggressive properties of glioma. This discrepancy can be addressed by developing complex heterotypic models that include additional cellular components of the TME.

In order to comprehend the function of diverse cell types within the glioma microenvironment in their interaction with tumor cells, a 3D spheroid model was developed by co-culturing either astrocytes (CCF-STTG1 cell line), microglia (HMC3 cell line), or both cell types together with human glioma cells (U87MG cell line). The resulting spheroid models were characterized based on morphological (features of formation, spatial arrangement of cells, and their viability) and molecular (cancer-specific markers, extracellular matrix protein synthesis, cytokine and chemokine secretion) parameters involved in glioma progression.

## 2 Methods

### 2.1 Cell lines

The U87MG glioblastoma cell line was purchased from the American Type Culture Collection (ATCC, Manassas, VA, United States). The CCF-STTG1 astrocytoma cell line and the HMC3 human microglial cell line were obtained from the Sputnik International Archive of Biomaterials (SIAB, Moscow, Russia). U87MG cells were cultivated in Minimum Essential Medium (αMEM) (#3075927, ThermoFisher/Gibco, Paisley, United Kingdom). The CCF-STTG1 and HMC3 cell lines were maintained in DMEM/F12 (#42400028). All culture media were supplemented with 10% fetal bovine serum (FBS) (#A316040), GlutaMAX™ solution (#35050061), 250 mg/mL amphotericin B, and 100 U/mL penicillin/streptomycin (1× antibiotic-antimycotic solution, #15140122). The DMEM/F12 culture medium was further enriched with MEM non-essential amino acids solution (#11140050) and sodium pyruvate (#11360070, ThermoFisher/Gibco, New York, NY, United States). Cells were cultivated at 37 °C in a 5% CO2 atmosphere unless otherwise indicated. Upon reaching 70%–80% confluence, the cells were washed with 2 mL of 1× PBS (Dia-M, Moscow, Russia) and incubated with 400 μL of TrypLE™ solution (#12604013, Invitrogen, MA, United States) for 3–5 min at 37 °C. Following detachment, the cells were washed with 1 mL of culture medium, and an aliquot of the cell suspension was transferred to a new culture flask containing 5 mL of fresh culture medium.

### 2.2 Spheroid formation

The generation of homo- and heterotypic spheroids was accomplished through the utilization of the liquid overlay method, a technique that employs 96-well Nunclon™ Sphera™ plates with U-shaped bottoms (#174925, ThermoFisher, MA, USA) or 24-well Nunclon™ Sphera™ plates (#174930, ThermoFisher, MA, USA). In order to induce the process of spheroid formation, the cells were resuspended in 100 μL of 3D medium. This medium was composed of DMEM/F12, which was supplemented with 1× GlutaMAX™, 1× antibiotic-antimycotic solution, 20 ng/mL epidermal growth factor (EGF; #E9644, Sigma-Aldrich), 20 ng/mL basic fibroblast growth factor (bFGF; #PHG0261), 5 μg/mL insulin (#I9278, Sigma-Aldrich), 2% B27 Plus supplement (#A35828010, ThermoFisher/Gibco) and 1% N-2 Supplement (#17502048, ThermoFisher/Gibco) and 4% BSA fraction V (#126593, Sigma-Aldrich). The selection of this growth factor cocktail was made on the basis of previously published protocols (Leonteva et al., 2026; Leonteva et al., 2025). Homotypic 3D spheroids were generated from single cell types: glioblastoma (3DG), astrocytes (3DA), and microglia (3DM). The generation of heterotypic 3D co-culture models (3D-2GA) involved the suspension of astrocytes with glioblastoma cells at ratios of 1:1, 3:2, and 4:1. Analogous ratios were utilized for co-cultures of microglia with glioblastoma cells (3D-2GM), where the proportion of tumor cells was the lowest. Three-component models (3D-3) were obtained by simultaneous seeding of all 3 cell types at ratios of 3:2:2, 3:3:4, and 5:2:3 (glioblastoma cells: astrocytes: microglia). The cells were seeded at a density of 2,500 cells per well in 100 μL of 3D culture medium. The plates were then subjected to an incubation period of 72 h at a temperature of 37 °C in an atmosphere containing 5% CO2, with the objective of enabling aggregation and subsequent spheroid formation. The process was monitored using an inverted Nexcope NIB620 microscope (Novel Optics, China). Each experiment was performed in three independent replicates (n = 3). In order to assess growth kinetics, confocal microscopy and viability, 96-well plates were utilized. All experiments pertaining to the analysis of proteins (cytokines, flow cytometry and Western blot) were conducted within 24-well plates.

### 2.3 The estimation of spheroid volumes and the measurement of growth kinetics

Spheroids were generated according to established protocols. Spheroids were cultivated for a period of 7 days, and the dynamics of their growth were monitored on days 1–5 of the cultivation period. The acquisition of images was conducted using an Evos M5000 microscope (ThermoFisher Scientific), and subsequent processing was undertaken with the utilization of Fiji software (ImageJ 2.16.0/1.54p, Java 1.8.0_442 64-bit). The volume of each spheroid was calculated using the following formula: V is equivalent to 0.5 × L × W^2^, where L is defined as the diameter that connects the pair of farthest points on the spheroid contour, and W is the largest diameter perpendicular to L (Chen et al., 2014). This approach is widely used for quantitative assessment of tumor spheroid growth. Growth kinetics histograms were generated for each model type. Each experiment was performed in three independent replicates (n = 3) in 96-well Nunclon™ Sphera™ plates with U-shaped bottoms (#174925, ThermoFisher, MA, United States).

### 2.4 Time-lapse imaging of spheroid formation

The process of spheroid formation was closely observed and documented through the implementation of the CELENA X high-throughput imaging system (Logos Biosystems, Anyang-Si, South Korea). Prior to the seeding process, the cells were labeled with vital fluorescent dyes. The glioblastoma cell line was stained with CellTracker Green CMFDA, while the astrocytes were labeled with CellTracker Red CMTPX (#C34552, Invitrogen, Carlsbad, CA, USA). Finally, the microglia were stained with CytoTracer Blue CMAC (#4682-10mg, Lumiprobe, Moscow, Russia). The cells were then seeded into U-bottom, low-adhesion Nunclon™ Sphera™ plates, as previously outlined. The plate was subsequently placed into the incubation chamber of the CELENA X system, and time-lapse imaging was performed for each well at 15-min intervals over a 72-h period. Following the conclusion of the experiment, the images were compiled into video files. In order to perform a comparison of the kinetics of spheroid formation, the endpoint of the aggregation stage was selected as the reference time point. This endpoint was defined as the completion of cell and cellular aggregate fusion. Each experiment was performed in three independent replicates (n = 3) in 96-well Nunclon™ Sphera™ plates with U-shaped bottoms (#174925, ThermoFisher, MA, USA).

### 2.5 Confocal microscopy

To ascertain the cell localization within spheroids, fluorescently labeled U87MG glioblastoma cells expressing GFP, CCF-STTG1 astrocytoma cells expressing the red fluorescent protein mKate2, and HMC3 microglial cells stained with CytoTracer Blue CMAC were utilized. Spheroids were generated in accordance with the protocol previously delineated. Following a 24-h period, the spheroids were washed with 1× PBS, transferred to a flat-bottom plate (Eppendorf, Hamburg, Germany), and visualized using an LSM 980 confocal microscope equipped with an Airyscan system (Carl Zeiss, Jena, Germany). The analysis of images was conducted using Fiji software (ImageJ 2.16.0/1.54p, Java 1.8.0_442, 64-bit). Each experiment was performed in three independent replicates (n = 3) in 96-well Nunclon™ Sphera™ plates with U-shaped bottoms (#174925, ThermoFisher, MA, USA). Fluorescence intensity-based mapping in different Z-stacks was analyzed with ZEN 3.4 Blue Edition (Carl Zeiss Microscopy GmbH, Jena, Germany) using the “profile” option to determine the area of signal of a different type of cell in a spheroid. By drawing a line across regions of interest (ROI), generated graphs mapping signal intensity against distance.

### 2.6 Penetration of FITC-Dextran into spheroids

On day three of the culture process, the spheroids were incubated with 200 μg/mL of FITC-Dextran 70 (#90718, Sigma-Aldrich). After 2 days, the spheroids were washed with 1× PBS, transferred to a flat-bottom plate (Eppendorf, Hamburg, Germany), and visualized using an LSM 980 confocal microscope equipped with an Airyscan system (Carl Zeiss, Oberkochen, Germany). The images were analyzed using Fiji software (ImageJ 2.16.0/1.54p, Java 1.8.0_442, 64-bit). Each experiment was performed in three independent replicates (n = 3) in 96-well Nunclon™ Sphera™ plates with U-shaped bottoms (#174925, ThermoFisher, MA, United States).

### 2.7 Cell viability assessment

Cell viability was assessed on days 3, 5, and 7 of culture. The formed spheroids were stained with 1 μg/mL FDA (#F1303, ThermoFisher), diluted in DMEM/F12 without FBS, for 30 min at 37 °C. After removing the FDA and washing with 1× PBS, the spheroids were stained with 5 μg/mL PI (BD Biosciences, NJ, USA) and Hoechst 33342 at a dilution of 1:1,000 (#2G010, Lumiprobe, Moscow, Russia) in PBS for 5 min at 37 °C. The spheroids were then washed with 1× PBS, transferred to a 96-well flat-bottom plate (Eppendorf), and visualized using an EVOS M5000 microscope (ThermoFisher Scientific). Images were analyzed using Fiji software (ImageJ 2.16.0/1/54p, Java 1.8.0_442 (64-bit)). Each experiment was performed in three independent replicates (n = 3) in 96-well Nunclon™ Sphera™ plates with U-shaped bottoms (#174925, ThermoFisher, MA, United States).

### 2.8 Assessment of metabolic activity (ATP)

The metabolic activity of the spheroids was evaluated on day 5 using the CellTiter-Glo® 2.0 Assay Kit (#G9241, Promega Corporation, Madison, WI, USA). The spheroids were transferred to black 96-well plates (Sovtech, Russia) in 100 μL of culture medium. Then, 100 μL of CellTiter-Glo® luminescent reagent was added. The plates were then incubated at room temperature: 2 min on an orbital shaker, followed by a 10-min incubation without shaking. Luminescence was measured in relative luminescence units (RLU) using a CLARIOstar microplate reader (BMG LabTech, Ortenberg, Germany) with an integration time of 1 s. To convert the signal to absolute ATP concentrations, a calibration curve was generated using ATP standards in the range of 1000-0.0001nM (1μM, 100nM, 10nM, 1nM, 0.1nM, 0.01nM, 0.0001nM). All experiments were performed in three to five biological replicates in 96-well Nunclon™ Sphera™ plates with U-shaped bottoms (#174925, ThermoFisher, MA, United States).

### 2.9 Assessment of mitochondrial membrane potential

The mitochondrial membrane potential (ΔΨm) was evaluated using the voltage-sensitive dye LumiTracker® Mito JC-1 (#2275-1mg, Lumiprobe). The cells were incubated with a 5 μg/mL working solution of the dye in the dark at 37 °C for 15 min, followed by washing with 1× PBS. Visualization was performed using a LSM 980 confocal microscope (Carl Zeiss). JC-1 monomers were excited with a 488-nm laser, and emission was recorded in the range of 500–540 nm. J-aggregates were excited using a 561-nm laser, and emission was recorded in the range of 570–620 nm.

### 2.10 Multiplex secretome analysis

The concentrations of cytokines and chemokines secreted into the culture medium by spheroids were determined using xMAP-based technology with the Bio-Plex Pro Human Cytokine Screening 48-Plex Panel (#12007283, Bio-Rad Laboratories, Hercules, California, United States) according to the manufacturer’s recommendations. Briefly, lyophilized standards were reconstituted in the provided standard diluent and serially diluted to generate a seven-point standard curve for each analyte. Spheroids were formed in 24-well low-adhesion plates at a density of 200,000 cells per well to obtain sufficient material. On day 5 of culture, the contents of the wells were transferred to centrifuge tubes. Then, bovine serum albumin (BSA) was added to a final concentration of 0.5%, and the tubes were centrifuged at 1,000 × g for 15 min at 4 °C to pellet the spheroids and cellular debris. The supernatant was then filtered through a 0.22-μm membrane and incubated with antibody-coupled magnetic capture beads for 16 h at 4 °C. After washing, detection antibodies were added and incubated for 30 min, followed by streptavidin-phycoerythrin incubation for 10 min. All incubation steps were performed with continuous shaking at 850 rpm. Measurements were performed on a QuattroPlex Lab Analyzer (Dia-M, Moscow, Russia) - a Luminex-based multiplex platform - according to the manufacturer’s instructions. The median fluorescence intensity (MFI) for each bead region was recorded, and analyte concentrations (pg/mL) were calculated from standard curves using QuattroPlex Lab software (v.1.5.3). Each sample was analyzed in triplicate. For analytes with concentrations below the lower limit of quantification (LLOQ), a value of LLOQ/2 was assigned for statistical analysis. The data were presented as absolute concentrations in pg/mL since equal numbers of cells were seeded for spheroid formation and spheroid size did not differ significantly between conditions at the time of collection.

### 2.11 Flow cytometry analysis

All flow cytometry analyses were performed using a FACSCanto II flow cytometer (BD Biosciences, Franklin Lakes, NJ, USA), and the data were processed using FACSDiva software, v.6.1.3 (BD Biosciences). Spheroid formation was carried out according to an established protocol previously described in 24-well Nunclon™ Sphera™ plates (#174930, ThermoFisher, MA, United States). Remove debris by gating on the main cell population in Forward Scattered Light (FSC) vs. Side Scattered Light (SSC). Remove doublets by gating on single cells using FSC (Area) vs. FSC (Height). Gate for live cells by gating on the live/dead low population. Initial cell gating was based on forward and side scatter to exclude debris, and 10,000 events were collected from the selected population (Cossarizza et al., 2019). The following antibodies were used for analysis: anti-CD44-APC and anti-CD24-PECy7 (#560890 and #561646, respectively, BD Pharmingen, San Diego, CA, United States) and anti-GFAP-FITC and anti-CD133-APC (#53–9792-82 and #17-1338-42, respectively, Invitrogen, Carlsbad, CA, United States). Compensation was performed on single stained beads (#01-2222-42, UltraComp eBeads™ Compensation Beads, ThermoFisher Scientific).

Spheroids were harvested, washed twice with 1× PBS, and dissociated using 500 μL of Accutase® (Capricorn, Ebsdorfergrund, Germany) for 10 min at 37 °C in 5% CO2, unless otherwise specified. To facilitate mechanical-enzymatic dissociation, the suspension underwent 10 up-and-down pipetting cycles. Then, a serum-containing medium was added to neutralize the Accutase®, and the cells were centrifuged at 1000 rpm for 5 min. The cell suspension was washed with 800 μL of PBS containing 2% FBS, filtered through a 70-μm cell strainer (BD Biosciences), and centrifuged again at 1,000 rpm for 5 min. After cell enrichment, the cells were stained with an antibody cocktail and analyzed by flow cytometry. Prior to analysis, NucBlue™ Live ReadyProbes™ (#R37605, Invitrogen) was added to the cell suspension. Complete spheroid dissociation was verified, and single-cell counts were determined using a LUNA-II™ automated cell counter.

### 2.12 Western blotting

Spheroids were generated according to the protocol described in Section 2.2 in 24-well Nunclon™ Sphera™ plates (#174930, ThermoFisher, MA, United States). On day 5 of culture, the spheroids from each well were transferred to centrifuge tubes. The spheroids were pelleted by centrifugation at 1000 *g* for 15 min at 4 °C. The pellet was then lysed in RIPA buffer (150 mM NaCl, 1% Triton X-100, 0.1% SDS, and 50 mM Tris-HCl, pH 7.4), which was supplemented with a 1× protease inhibitor cocktail (#G2006-250UL, ServiceBio, Wuhan, China). The protein concentration was determined using the Bradford protein assay kit (#K002, FineTest, Wuhan, China). The protein lysates were loaded onto 4%–20% SDS-PAGE gels (#PSG 2001-415T, WSHT, Shanghai, China) with 4× loading buffer (#D-Prot-ME-01, Biolabmix, Novosibirsk, Russia). Following electrophoresis, the proteins were transferred onto PVDF (#1620174, Bio-Rad, Hercules, CA, United States) using wet electrotransfer. The membranes were blocked with 5% nonfat milk (#3905.0500, Dia-M, Moscow, Russia) at room temperature for 1 h, followed by an overnight incubation at 4 °C with primary antibodies (ABclonal, Wuhan, China) against the following targets: SNAIL (1:1000, #A5544), SLUG (1:1000, #A27232), TWIST (1:1000, #A3237), HIF1β (1:1.000, #A19532), vimentin (1:1000, #A19607), VEGF (1:1000, #A23759), collagen I (1:1000, #A24112), collagen IV (1:1000, #A25916), fibronectin (1:1000, #A16678), laminin (1:1000, #A25510), MMP-2 (1:1000, #A6247), MMP-9 (1:1000, #A0289), MMP-10 (1:1000, #A3033), and GAPDH (1:1000, #A19056). The membranes were five-times washed with Tris-buffered saline containing 0.1% Tween-20 (TBST) for 30 min, and then incubated for 1 hour at room temperature with constant agitation using horseradish peroxidase-conjugated anti-rabbit secondary antibodies (1:10000, ABclonal). Membranes were washed five times with TBST. Signals were detected using a chemiluminescent substrate (#G2074-50ML, ServiceBio, Wuhan, China) on a ChemiDoc imaging system (Bio-Rad, Hercules, CA). Images were processed using Image Lab software (Bio-Rad, Hercules, CA, United States).

### 2.13 Assessment of the invasive and migration potential of cells within spheroids

Migration and invasion are two clearly separated terms in experimental cell biology (Kramer et al., 2013). In order to assess cell migration abilities *in vitro*, 2% gelatin can be used as a substrate, as it is a porous, inert material in which tumor cells are able to spread unhindered. One of the classical methods to study cell on-top invasion *in vitro* is the test in substrate from Matrigel™. To evaluate the invasive activity of cells in 3D models, on day three, the spheroids were transferred to wells of a 96-well plate (TPP Techno Plastic Products AG, Trasadingen, Switzerland) that was coated with either a 50% Matrigel™ hydrogel substrate in PBS (BD Biosciences, San Jose, CA, USA) or cold-water fish skin 2% gelatin in PBS (Sigma, Oakville, ON, Canada). The hydrogel polymerized by incubating the plate at 37.0 °C ± 1.0 °C for 30 min. The spheroids were then placed on the hydrogel-coated substrate and cultured under standard conditions.

Invasion on gelatin was monitored for 3 days, while invasion on Matrigel™ was monitored for 8 days. Daily photo-documentation was performed using an EVOS M5000 microscope (ThermoFisher, MA, USA). Image analysis was performed using Fiji software (ImageJ 2.16.0/1.54p, Java 1.8.0_442 (64-bit)). To quantitatively assess cell motility on gelatin, we measured the spheroid radius. For the evaluation of Matrigel™, we measured the area occupied by cells. Measurements were performed immediately after transfer to the substrate and then daily. Each experiment was performed in three independent replicates (n = 3) in 96-well Nunclon™ Sphera™ plates with U-shaped bottoms (#174925, ThermoFisher, MA, United States).

### 2.14 Temozolomide treatment and cytotoxicity assessment

#### 2.14.1 2D model

Temozolomide (TMZ; Temodal®, Orion Corporation, Orion Pharma, Finland) was used in this study. TMZ was dissolved in sterile water for injection to prepare a stock concentration of 6 mM active substance and then diluted to the required concentrations with a complete cell culture medium. For drug treatment, glioma cells were seeded in 96-well plates (5 × 10^4^ per well) and cultured for 24 h. For the MTT-assay, TMZ was added to the cells at concentrations 100, 200, 300, 400, 500, and 1000 µM. Untreated cells served as controls. In the present study, the cultures were exposed to TMZ for 48 h for U87 MG or 72 h for all cell lines. After 48 and 72 h of treatment, metabolic activity was assessed using the MTT assay according to the manufacturer’s protocol (#M2128-5G, Sigma Oakville, ON, Canada), at 570 nm, with a reference wavelength of 620 nm. Absorbance was measured using a CLARIOstar microplate reader (BMG LabTech, Ortenberg, Germany). Cell viability was determined relative to control cell viability (100%) ± standard deviation from three independent experiments. IC50 was calculated using CompuSyn software, v.1.4 (ComboSyn, Inc., Paramus, NJ, United States).

#### 2.14.2 3D model

Spheroids in four independent replicates (n = 4) were generated according to the protocol described in Section 2.2 in 96-well Nunclon™ Sphera™ plates with U-shaped bottoms (#174925, ThermoFisher, MA, United States). On the third day of spheroid culture, TMZ was added at concentrations of 100 and 1000 μM. The drug’s cytotoxic effect was then assessed using the MTT assay after 48 and 72 h. Furthermore, 72 h following TMZ exposure, the volume of the spheroids and cell viability were evaluated using the methodologies outlined in Sections 2.7 and 2.3, respectively. Images were acquired with a fluorescence microscope using an EVOS M5000 microscope (ThermoFisher, MA, United States) and analyzed using Fiji software (ImageJ 2.16.0/1.54p, Java 1.8.0_442 (64-bit)). Fluorescence intensity-based mapping was analyzed with ZEN 3.4 Blue Edition (Carl Zeiss Microscopy GmbH, Jena, Germany) using the “profile” option to determine the area of signal of live and dead cells in a spheroid. By drawing a line across regions of interest (ROI), generated graphs mapping signal intensity against distance. Mean intensities are divided by background noise to calculate a normalized signal, correcting for baseline auto-fluorescence. Finally, the relative ratio of living (FDA-positive) and dead (PI-positive) cells is normalized against the total nuclear signal (Hoechst 33342-positive). Additionally, the ratio of changes in the volume of spheroids before and after sample preparation was evaluated in order to distinguish live and dead cells (k-parameter V_pre/V_post). The volumes V_pre and V_post are defined as the volumes prior to and subsequent to staining, respectively. In order to analyze the effect of the drug, the total cell density (live or dead cells) was calculated, with the ratio of spheroid volume change (live: (FDA+/Hoechst 33342+)/k) or (dead: (PI+/Hoechst 33342+)/k) being taken into account. Following the acquisition of the relevant data (viability index), the construction of histograms was undertaken, and a statistical analysis was subsequently conducted.

### 2.15 Statistical analysis

Statistical analysis was performed using Prism v.10.3 software (GraphPad Software, San Diego, CA, United States). All data are presented as the arithmetic mean ± standard deviation (M ± SD). One-way and two-way analyses of variance (ANOVAs) were applied to compare groups, followed by Tukey’s multiple comparisons test. Differences were considered statistically significant at the following levels: *p < 0.05, **p < 0.01, ***p < 0.001, and ****p < 0.0001. All experiments were performed with at least three to five biological replicates. To assess the relationships between cytokine concentrations, we calculated Pearson’s linear correlation coefficients (r). The normality of the quantitative variable distribution was preliminarily verified using the Shapiro-Wilk test (p > 0.05). Based on the obtained coefficients, a correlation matrix was constructed and visualized as a heat-map without additional scaling or data normalization. The critical significance level was set at 0.05.

## 3 Results

### 3.1 Characteristics of the formation and growth of 3D spheroid brain tumors models

**FIGURE 1:**
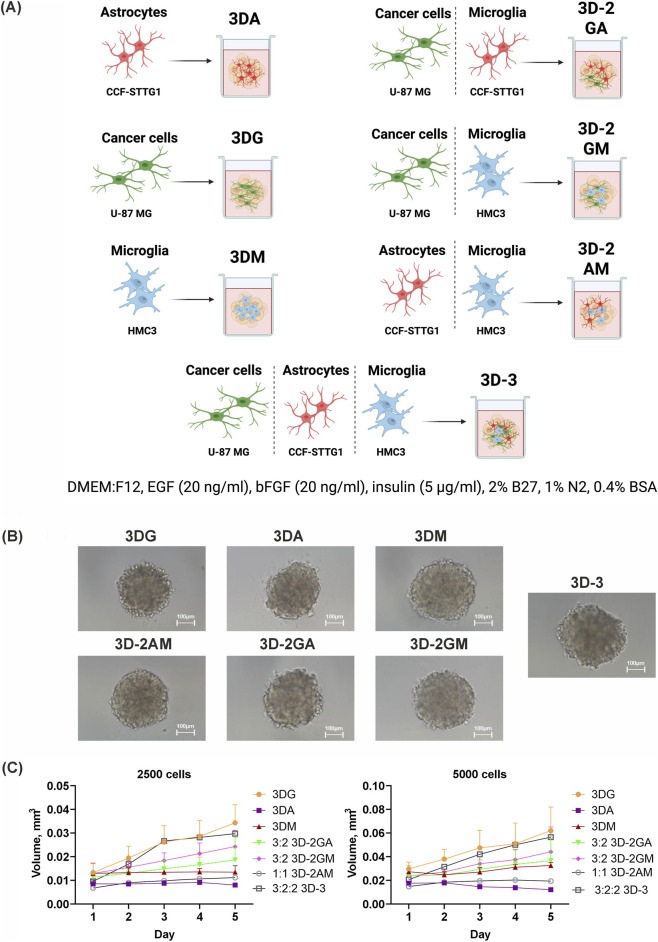
Homotypic and heterotypic spheroids of different brain tumor models. **(A)** Schematic representation of the formation of 3D glioma spheroids composed of CCF-STTG1 astrocytes, HMC3 microglia, and U87MG glioma, depicting co-culture of cells in 96-well U-bottom plates for 3–5 days to form stable and mature 3D glioma spheroids. **(B)** Representative images of 3D brain tumor cell models. Data for 24 h of incubation are shown. **(C)** Dynamics of changes in spheroid model volume depending on cell concentration and cellular composition of the 3D model.

According to histopathological studies and flow cytometry, biopsies of human and mouse gliomas consist of 30%–40% microglia (Matias et al., 2018; Annovazzi et al., 2018; Caruso et al., 2020). Concurrently, astrocytes account for nearly 30% of all cells in the human brain (Zhang et al., 2023). In the development of heterotypic models, it is imperative to consider not only this ratio but also the initial rate of cell proliferation in culture. The approach to forming a complex heterotypic model involves the mixing of U-87MG glioma tumor cells with CCF-STTG1 astrocytes and HMC3 microglia in a specific ratio (Figure 1C; Supplementary Figure S1). These cells are then seeded into wells of a low-adhesion 96-well Nunclon™ Sphera™ plate under standard conditions in serum-free DMEM/F-12 medium supplemented with growth factors (Figure 1A) (Leonteva et al., 2026; Leonteva et al., 2025). Thus, brain tumor spheroids were cultivated under the following conditions: the 3D monoculture model involves the formation of a spheroid exclusively from tumor cells (hereinafter 3DG), or from astrocytes (hereinafter 3DA), or from microglia (hereinafter 3DM). The co-cultivation of tumor cells and astrocytes (hereinafter 3D-2GA), or tumor cells and microglia (hereinafter 3D-2GM), or astrocytes and microglia (hereinafter 3D-2AM), and tumor cells, astrocytes, and microglia (3D-3) (Figure 1B) are also examined.

Irrespective of the number of cells utilized in the formation of the spheroid, an augmentation in the volume of 3DG models was evident over a 5-day period. Conversely, the volume of the 3DA spheroid did not exhibit an increase, maintaining a compact configuration (Figure 1C; Supplementary Figure S1). A comparative analysis of changes in the volume of 3D-2GA spheroids demonstrated that the model with the highest proportion of astrocytes (4:1) exhibited the least change, in contrast to models containing a higher proportion of tumor cells (1:1 and 3:2). A similar trend was observed when evaluating changes in the size of the 3D-2GM model (Figure 1C; Supplementary Figure S1). As the number of microglia (4:1) in the model increased, the volume of the spheroid model changed less. The volume of the 3D-2AM spheroid model (1:1) remains constant, and the model maintains a compact form. For the complex 3D-3 model, the model volume increased with the addition of microenvironment cells, though it remained smaller than that of the 3DG model (Figure 1C; Supplementary Figure S1).

**FIGURE 2:**
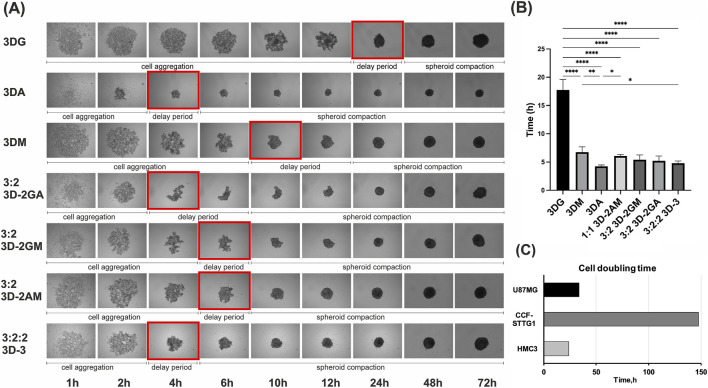
The process of homotypic and heterotypic spheroid formation simulating different 3D models of brain tumors. Time-lapse photography of the 3D, 3D-2 and 3D-3 models. The data show **(A)** stages and **(B)** time of spheroid formation; **(C)** cell doubling time of U87MG, CCF-STTG1, HMC3. Data are presented as mean values (M) with standard deviation (SD), calculated from triplets of independent measurements. The difference between the experimental groups was statistically significant at *p < 0.05; **p < 0.01; ***p < 0.001; ****p < 0.0001 (one-way ANOVA with Shapiro-Wilk test).

**FIGURE 3:**
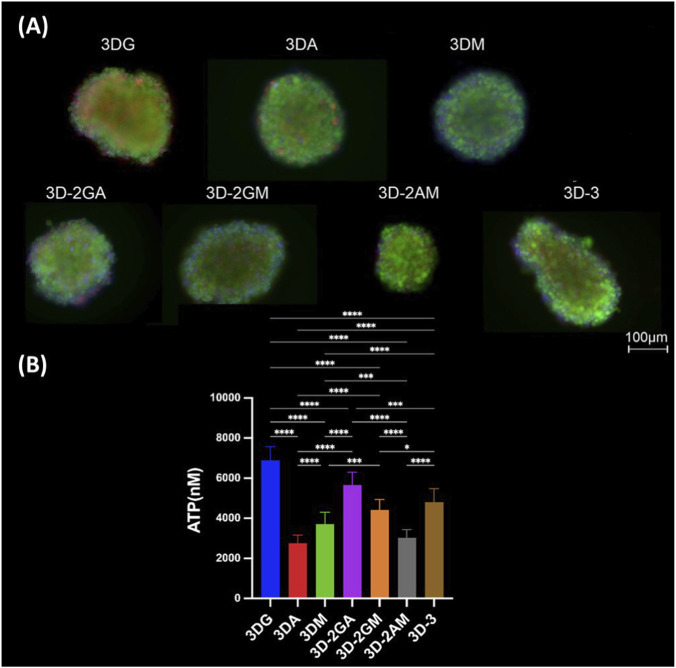
Cell viability analysis in homotypic and heterotypic 3D models. **(A)** Intravital cell viability assessment. Green fluorescent signal is the FDA vital cell dye, red fluorescent signal is the PI dead cell dye, and blue fluorescent signal is the Hoechst 33342 cell nuclear dye. The scale bar is 100 µm. **(B)** Analysis of ATP content in cells of heterotypic 3D brain tumor models. Total intracellular ATP content was measured by luminescence using the CellTiter-Glo ® Cell Viability Assay (Promega). Data are the average values from three independent measurements.

During the process of aggregation, cells undergo fusion, resulting in the formation of numerous amorphous cell aggregates. These aggregates subsequently undergo a process of rounding, ultimately leading to the formation of spheroids. The cells coalesce into amorphous cellular aggregates and subsequently transition to the compaction stage, characterized by the formation of dense spheroids, a process that is time-dependent (Figure 2; Supplementary Figure S1D). No correlation was observed between the time of spheroid formation and the doubling time of the cells forming the spheroids. HMC3 > U-87MG > CCF-STTG1 (Figure 2C). During the process of forming homotypic spheroids from U-87MG, cells aggregated with each other within the first 16 h and formed a spheroid after 24 h, followed by compaction of the model. 3DA and 3DM spheroids were formed within 4 and 8 h, respectively (Figure 2). 3D-2GA spheroid models in a 1:1 and 3:2 ratio formed on average within 6 h, in contrast to the 4:1 ratio, where the spheroid formed within 12 h. For 3D-2GM and 3D-2AM, irrespective of the cell ratio in the model, the formation of spheroids typically occurred within 6 h (Figure 2; Supplementary Figure S1). The 3D-3 model exhibited a propensity for accelerated spheroid formation as the number of added astrocytes increased; however, on average, the model formed within 6 h, after which it underwent further growth and consolidation (Figure 2; Supplementary Figure S1). After determining the optimal ratio and cell viability in the model, the following cell ratios were selected for cultivation in subsequent experiments: for 3D-2GA, 3D-2GM - 3:2, for 3D-2AM - 1:1, and for 3D-3 - 3:2:2 (Figure 1C; Supplementary Figure S1). Cells in the selected ratios demonstrated viability for 7 days of culture without the apparent formation of a necrotic nucleus (Figure 3A).

ATP (adenosine triphosphate) functions as a pivotal signaling molecule and energy source in glioma, promoting rapid tumor growth, invasion, and resistance to therapy (Wu et al., 2024; Yang et al., 2025). The ATP levels in spheroids exhibited a sequential order, with 3DG having the highest level, followed by 3D-2GA, 3D-3, 3DM, 3D-2GM, 3D-2AM, and 3DA, as depicted in Figure 3B. This assay has been shown to correlate with alterations in the volume of the spheroids (Figure 1C). The 3DG model demonstrates maximum ATP levels, indicative of their aggressive, proliferative phenotype. The incorporation of TME cells (astrocytes and microglia) within the spheroid has been demonstrated to result in a reduction of the total ATP pool. This phenomenon may be attributed to two factors: first, competition for nutrients, and second, the transition of TME cells to an activated state, which necessitates additional energy expenditure. The presence of low ATP levels is a characteristic feature of 3DA. Astrocytes with low basal ATP levels exhibit metabolic dysfunction, particularly in the TME, where they undergo reprogramming to sustain tumor progression. JC-1 staining was performed to assess the condition of the mitochondria and their membrane potential (Supplementary Figure S2). The reduced JC-1 level is indicative of mitochondrial depolarization in U-87MG cells and is consistent with established data on the predominant use of glycolysis by U-87MG cells for energy production. Elevated JC-1 levels in the CCF-STTG1 culture are indicative of hyperpolarization of mitochondrial membranes, accompanied by low ATP levels during 3D culture. This phenomenon may be attributable to either active oxidative metabolism or mitochondrial stress with impaired ATP synthesis. Healthy HMC3 microglial cells are characterized by a balanced mitochondrial potential.

Therefore, the study of spheroid growth dynamics demonstrated that the homotypic model derived from glioma cells is characterized by the largest spheroid size, irrespective of the total number of cells in the model. However, this model exhibits a slower rate of spheroid aggregation and compaction. The co-culturing of tumor cells with TME cells has been demonstrated to promote aggregation and the formation of a viable complex heterotopic spheroid model, with astrocytes contributing more to this process than microglia. The alterations in baseline ATP levels mirror the reliance of the energy status of multicellular tumor spheroids on the composition of their microenvironment.

### 3.2 Assessment of cell localization in 3D spheroid brain tumors models

**FIGURE 4:**
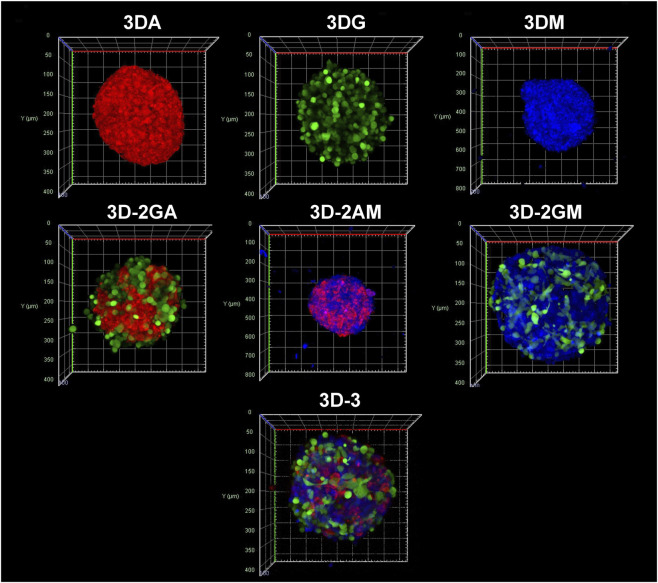
Analysis of intercellular interactions in homotypic and heterotypic spheroid models. U-87MG glioma cells (green), CCF-STTG1 microglia (blue), and CCF-STTG1 astrocytes (red). Confocal microscopy, scale bar 100 μm.

The cellular composition and distribution of cells on the developed platform of homo- and heterotypic spheroids were analyzed using confocal microscopy after a 24-h period (Figure 4). The intensity analysis along the center line of the spheroid is displayed in Supplementary Figures S3, S4. Conversely, 3DG resulted in less compact spheroids. Conversely, 3DA and 3DG exhibited enhanced compactness. In the case of 3D-2GA, the formation of a dense core of astrocyte-like cells was observed in the center of the spheroid, with glioma cells surrounding it. Consequently, astrocytes are able to establish a more robust core structure, while glioma cells organize in a peripheral configuration. In contrast to the 3D-2GA model, the 3D-2GM model does not exhibit the formation of a nucleus composed of a single cell type. In the case of 3D-2GM, the cells within the spheroid underwent spatial self-organization driven by homotypic intercellular interactions. By day 1 of co-culture, the formation of small clusters of astrocytes surrounded by microglial cells was observed. During the formation of the 3D-3 model, by day 1 the cells within the model were arranged in a disorderly manner, forming a heterogeneous structure (Figure 4).

**FIGURE 5:**
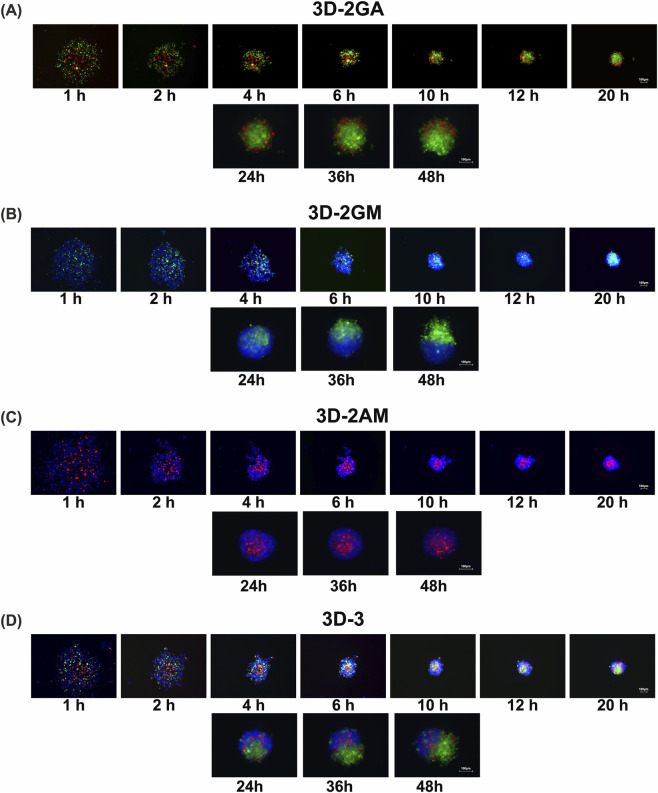
Changes in cellular rearrangement during the formation of heterotypic spheroid models. Time-lapse photography of the 3D-2 and 3D-3 models formation. **(A)** Relative dispositions of astrocyte-like cells CCF-STTG1 and glioma U-87MG in 3D-2GA depending on time. **(B)** Dispositions of microglia HMC3 and glioma U-87MG in 3D-2GM depending on the spheroid lifetime. **(C)** Localization of astrocyte-like cells CCF-STTG1 and microglia HMC3 in 3D-2AM. **(D)** Changes in the relative positions of microglia HMC3, astrocyte-like cells CCF-STTG1 and glioma U-87MG in 3D-3 model. U-87MG glioma cells–green fluorescent signal, CCF-STTG1 astrocyte-like cells–red fluorescent signal, HMC3 microglia cells–blue fluorescent signal.

A salient feature of spheroids is their capacity to attain high density and compactness, primarily attributable to cell rearrangement and the synthesis of their own extracellular matrix (Cordeiro et al., 2024). Real-time analysis of cell formation dynamics and spatial arrangement in a 3D-2GA model over 48 h revealed that during the first 6 h, astrocytes, possessing a high degree of adhesion, occupied a central position within the spheroid, forming the scaffold of the 3D model. Concurrently, glioma cells, characterized by their high degree of proliferation and invasion, occupied a peripheral position. However, a subsequent inversion of cell positions occurred in the model, resulting in the formation of an inner core by glioma cells and the accumulation of astrocytes at the periphery of the spheroid (Figure 5A). In the 3D-2GM model, an inner core of tumor cells is formed during the first day, followed by pronounced cell polarization within the spheroid, with the formation of two distinct poles within 24–48 h (Figure 5B). In the 3D-2AM model, astrocytes are located centrally, and the formation of microclusters of various sizes is observed, which begin to interact with one another and form the inner core of the spheroid. By the end of the second day, astrocyte microclusters begin to shift toward one of the spheroid’s poles. However, in contrast to the 3D-2GM models, no formation of clearly distinguishable poles is observed (Figure 5C). During the formation of a complete 3D-3 model, microglia are located at the periphery, while tumor cells and astrocytes are found at the center. By the second day, the tumor and microglial poles are clearly distinguishable, while astrocytes are diffusely distributed, forming a branched supporting network (Figure 5D). A video illustrating cell formation and localization in 3D cell cultures is presented in the supplementary material (Supplementary Figure S5). Microglia are cells that exhibit high levels of mobility; they are capable of actively migrating along oxygen and nutrient gradients toward the surface of the spheroid, where conditions are more favorable (Li and Graeber, 2012; Tao et al., 2023). Tumor cells characteristically exhibit reduced mobility and form compact clusters.

Consequently, the selected conditions promote the formation of dense heterotypic brain tumor spheroids, wherein astrocytes function as a supportive framework, while microglia and tumor cells are spatially segregated into two distinct segments. This spatial configuration of cells gives rise to a complex and localized aggressive microenvironment *in vitro*. To elucidate the mechanisms underlying its aggressiveness, an analysis of the cytokine profile was conducted, as cytokines are the key effectors of intercellular communication in the TME.

### 3.3 Cytokine expression by cells in heterotypic 3D spheroid models

**FIGURE 6:**
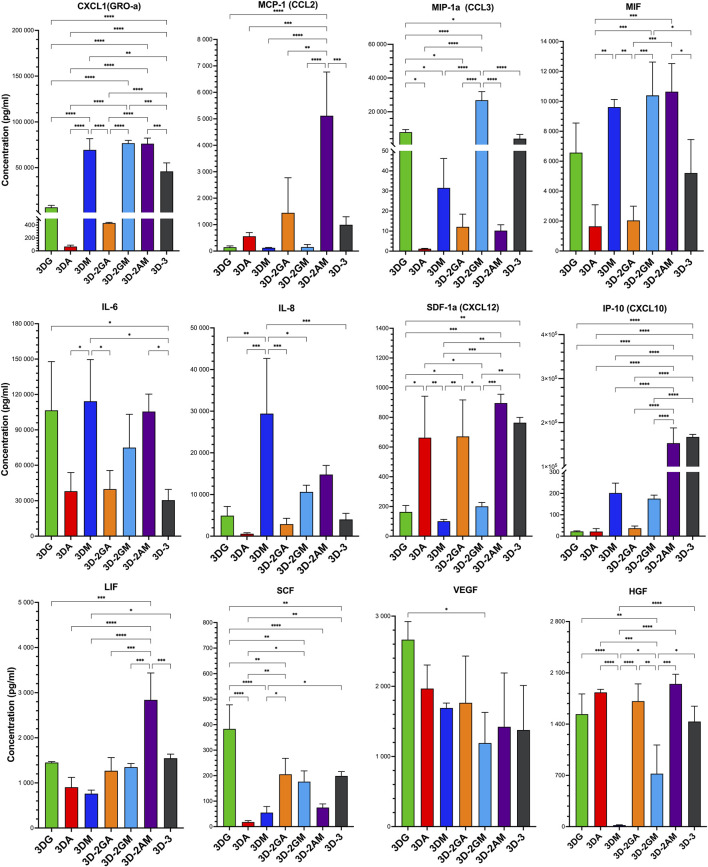
Concentrations of the selected cytokines in conditioned medium. Data are presented as mean values (M) with standard deviation (SD), calculated from triplets of independent measurements for each cytokine. The difference between the experimental groups was statistically significant at *p < 0.05; **p < 0.01; ***p < 0.001; ****p < 0.0001 (two-way ANOVA with Shapiro-Wilk test).

A multiplex analysis of a 48-cytokine/chemokine panel was conducted via the xMAP-based method to assess the influence of the cellular TME on cytokine secretion in 3D models. A comparison of 3D with their 3D-2 and 3D-3 variants revealed a number of non-additive effects, suggesting complex paracrine intercellular interactions. The TME of glioma is known to be characterized by high infiltration of immunosuppressive myeloid cells, whose recruitment is controlled by specific chemokines, including MCP-1 (CCL2), MIP-1α (CCL3), and LIF (Matias et al., 2018; Borisov and Sakaeva, 2015). The 3D-2AM model demonstrated a heightened level of MCP-1 secretion, which exceeded the combined contribution of the 3DA and 3DM by more than 10-fold (Figure 6). A notable observation was the substantial suppression of MCP-1 production in the 3D-3, indicating the capacity of glioma cells to actively regulate the chemokine signal, which may contribute to shaping the immune landscape of the model. A parallel increase in MIP-1α levels was observed in the 3D-2GM, where these levels exceeded those seen in 3DG and 3DM. In the 3D-3, this effect was negated, apparently due to the presence of astrocytes. LIF production, a known factor in maintaining glioma stemness and an inducer of immunosuppression, reached its maximum levels in heterotypic cultures containing astrocytes and microglia, peaking in the 3D-2AM culture, which was nearly twice as high as in the 3DG.

Inflammation, a hallmark of glioma progression, was characterized by elevated levels of IL-6, IL-8, GRO-α (CXCL1), and IP-10 (CXCL10) secretion (Matias et al., 2018). In 3DM and 3DG cultures, a marked increase in the production of IL-6 and IL-8 was observed, which was significantly suppressed in the presence of astrocytes. In 3D-2GA and, particularly, in 3D-3, the levels of IL-6 and IL-8 were reduced by 2.5–3.5-fold compared to 3D-2DG and 3D-2DM. This finding suggests that astrocytes may possess a suppressive capacity against pro-inflammatory stimuli within the TME. In all heterotypic models containing microglia, elevated GRO-α production was detected, thereby confirming the pivotal role of microglia in maintaining the pro-inflammatory and pro-angiogenic chemokine gradient. An unanticipated observation was the pronounced hyper-production of IP-10, which was discerned exclusively in cultures comprising both astrocytes and microglia. In all other models, IP-10 levels remained minimal. This finding suggests a cooperative interaction between astrocytes and microglia, resulting in the generation of IP-10, a well-known chemotactic factor for the recruitment of activated T-lymphocytes. Notably, this signal persists even in the presence of tumor cells, underscoring the significance of this cooperative interaction in the immune response.

Invasive growth and neoangiogenesis are hallmarks of glioma, as reflected in the secretion of vascular endothelial growth factor (VEGF), hepatocyte growth factor (HGF), and stromal cell-derived factor 1α (CXCL12). In the entirety of monotypic 3D, there was an elevation in VEGF levels that was comparable across cultures. However, a downward trend was observed in all heterotypic combinations, most pronounced in the 3D-2AM and 3D-3. This phenomenon may be indicative of regulatory feedback mechanisms operating within a complex microenvironment, which serve to impede excessive angiogenic signaling. The predominant producers of HGF were astrocytes and glioma cells. In contrast, microglia exhibited negligible HGF production. However, in the 3D-2GM, HGF levels paradoxically decreased by more than half compared to 3DM, indicating modulation of this invasion factor during tumor-immune cell interaction. The restoration of HGF levels in the 3D-3 underscores the modulatory role of astrocytes. The level of SDF-1α, a critical chemotactic factor directing the invasion of tumor cells and endothelial progenitors, was highest in the 3DA. Intriguingly, in heterotypic cultures comprising astrocytes co-cultured with microglia (3D-2AM) and/or glioma cells (3D-2GA, 3D-3), SDF-1α levels persisted at elevated levels, indicating that the microenvironment sustains and potentially amplifies the pro-invasive signal. HGF and SDF-1α have been identified as critical chemotactic factors that regulate the migration of tumor cells (via c-Met and CXCR4 receptors) and stem/progenitor cells. Their coordinated production in the microenvironment creates a “chemokine gradient” that promotes the invasion of tumor cells into the brain parenchyma and the formation of a perivascular niche. This HGF-induced activation of the SDF-1α axis and its CXCR4 receptor is one of the mechanisms stimulating tumor cell invasion (Tu et al., 2009). In light of the established role of this signaling axis in maintaining tumor stemness, the subsequent logical step in our research was to analyze markers associated with invasive potential and stemness.

### 3.4 Analysis of pro-invasive markers in heterotypic 3D spheroid models

**FIGURE 7:**
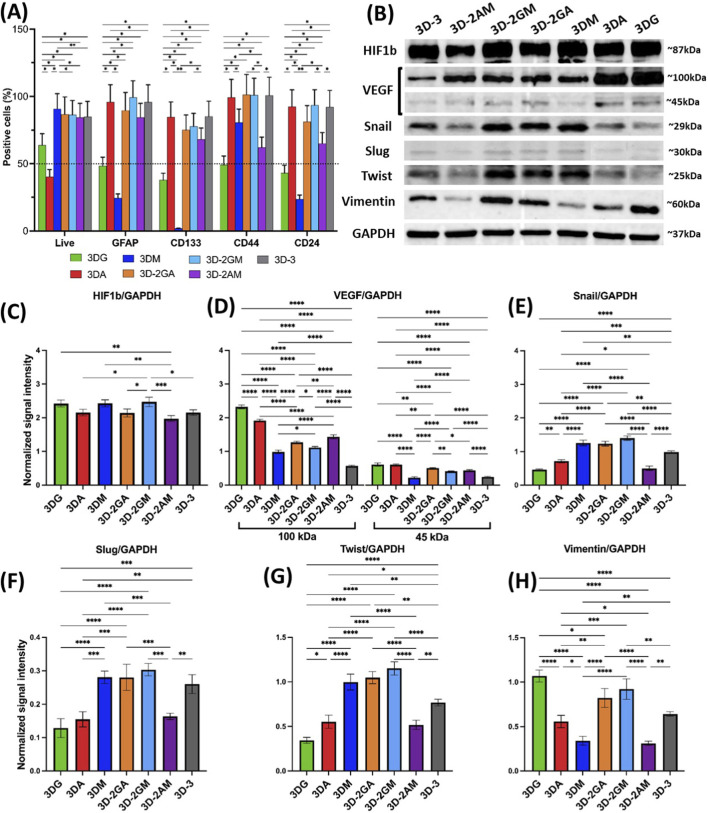
Molecular profiling of pro-invasive markers in homo- and heterotypic 3D brain tumor models. **(A)** Flow cytometry analysis of the CSC markers positive subpopulations in the spheroid model. Bar graph showing the percentage of GFAP+, CD133+, CD44^+^, CD24^+^ cells detected by flow cytometry. **(B)** The immunoblotting analyses of HIF1b **(C)**, VEGF **(D)**, SNAIL **(E)**, SLUG **(F)**, TWIST **(G)**, Vimentin **(H)** expression in 3D spheroids. One representative Western blot of two independent experiments is shown. Quantification of the protein expression was normalized to GAPDH as a loading control. The protein bands of the target were quantified as relative values to loading control bands. Data are presented as mean values (M) with standard deviation (SD), calculated from triplets of independent measurements. The difference between the experimental groups was statistically significant at *p < 0.05; **p < 0.01; ***p < 0.001; ****p < 0.0001 (one-way ANOVA with Shapiro-Wilk test).

Glial fibrillary acidic protein (GFAP) expression has been associated with astrocyte activation, and elevated levels in patients’ blood have been shown to indicate tumor progression (Jung et al., 2007). The presence of aggressive cancer stem cells (CSCs) within the glioma is a factor that contributes to glioma progression, recurrence, and drug resistance. When glioma CSCs are forced to differentiate, they typically undergo a loss of stem cell markers such as Nestin and Sox2 while increasing GFAP expression, transitioning from a stem-like state to a more glial-like tumor cell (Guichet et al., 2016; Pasqualetti et al., 2023). Among homotypic spheroid models, the proportion of GFAP-positive cells varied in the order 3DA > 3DG > 3DM (Figure 7A) and increased significantly in heterotypic spheroid models 3D-2GM compared to 3DG and 3DM. A possible source of GFAP expression in 3D cell cultures is neural stem cells supported by the presence of 1% N-2 Supplement in the medium. It should also be noted that GFAP level depends on the cell activation during the transition to a 3D cellular state, which may be associated with an increased level of cell stress with the formation of GFAP-positive tunneling nanotubes between cells (Simone et al., 2023). A subsequent analysis of the expression of key CSC proteins, CD133, CD44, and CD24, which drive glioma progression and increased invasive properties, revealed an increase in the proportion of cells positive for these markers in the presence of microenvironmental cells (Figure 7A).

Spheroid models are designed to mimic the physiological environment of solid tumors. Specifically, these models create an oxygen gradient that leads to the development of a hypoxic core. In conditions of hypoxia within the tumor, the HIF-1α and HIF-2α subunits are stabilized and dimerized with HIF-1β (also known as ARNT), thereby activating the transcription of VEGF. This, in turn, promotes tumor angiogenesis (Lee et al., 2019). Despite the elevated levels of HIF-1β protein observed in spheroid models, the levels of VEGF exhibit variability among the models examined (Figures 7B,C). This variability may be attributable to the presence of alternative signaling pathways that initiate VEGF transcription and post-translational processing, which are triggered by interactions between TME cells. A subsequent analysis of VEGF isoform expression revealed elevated levels in homotypic 3DG and 3DA spheroids, followed by a decline in VEGF levels in heterotypic spheroid models (Figures 7B,D).

Furthermore, the presence of CSCs has been shown to augment the invasive and metastatic potential of cells by epigenetically activating genes, such as SNAIL or TWIST, which are pivotal for the epithelial-mesenchymal transition (EMT) (Lee et al., 2025). The data on the differential expression of the transcription factors SNAIL, SLUG, and TWIST reflect the molecular plasticity of tumor cells when interacting with various components of the microenvironment (Figures 7B,E–G). A thorough analysis of SNAIL reveals elevated expression levels in 3DM, as well as in all heterotypic models, when contrasted with 3DG and 3DA. In glioma, this activity enhances the invasive potential of tumor cells through a coordinated decrease in adhesiveness and an increase in invasion. Emerging evidence suggests a potential role for SNAIL in modulating astrocytic responses to stimuli from glioma cells, facilitating the transition of astrocytes to a reactive state and the acquisition of EMT-like features (Iser et al., 2018). An interesting observation is the phenomenon of synergistic SNAIL induction in 3D-2GA, where individual expression in 3DG and 3DA is moderate/minimal, which may act as a trigger for the astrocyte-to-reactive astrocyte transition. In the 3D-2AM and 3D-3, an attenuation of the effect is observed: SNAIL expression is reduced relative to 3D-2GA/GM, which may reflect the influence of microglia modulating astrocyte paracrine signals. This finding aligns with existing data on the heterogeneity of the glial response, wherein microglia compete for signals that induce EMT. Another EMT protein, SLUG, exhibits a similar expression pattern to SNAIL, suggesting that it is differentially activated in various cellular contexts. It is noteworthy that SLUG and SNAIL function in parallel but can regulate distinct EMT programs depending on the specific type of cell. Furthermore, their expression may be contingent on particular microenvironmental signals (Xia, 2025). TWIST, yet another EMT protein, also exhibits a similar activation pattern to SLUG and SNAIL in both homotypic and heterotypic brain tumor models. Vimentin has been shown to form a feedback loop in which it is regulated by the TWIST protein. In turn, the TWIST protein maintains the expression of transcription factors such as SNAIL and SLUG, thereby promoting tumor progression (Parvanian and Eriksson, 2025). Among homotypic spheroid models, the highest vimentin protein level was detected in the 3DG model (Figures 7B,H). For heterotypic models, the 3D-2GA/GM model demonstrates a comparable protein level in contrast to the 3D-2AM model, indicating a more substantial contribution from tumor cells. The complex 3D-3 model demonstrates an intermediate level of vimentin protein.

Consequently, a meticulous and exhaustive evaluation of the pivotal markers of stem cell properties and invasiveness was conducted within the molecular profile of the 3D models. Given that the microenvironment is formed not only by cellular elements but also by extracellular matrix (ECM) synthesized by them, further analysis focused on studying the accumulation and deposition of ECM proteins in spheroid 3D models was undertaken.

### 3.5 Assessment of extracellular matrix protein accumulation and deposition in heterotypic 3D models

The extracellular matrix associated with brain tumors can be classified into three categories: tumor, normal brain, and the interface between the tumor and normal brain. The composition and tissue architecture of these three categories of ECM differ. It is widely accepted that the ECM of normal brain tissue is composed of four primary components: glycosaminoglycans, proteoglycans, hyaluronic acid, and adhesins, along with a modest amount of collagen. In contrast, the ECM of glioma contains elevated levels of collagen and hyaluronic acid, laminin, fibronectin, and hyaluronan. These factors contribute to chemoresistance, enhance stem cell properties, and promote glioma cell invasion through various molecular mechanisms (Marino et al., 2023; Wei et al., 2024).

**FIGURE 8:**
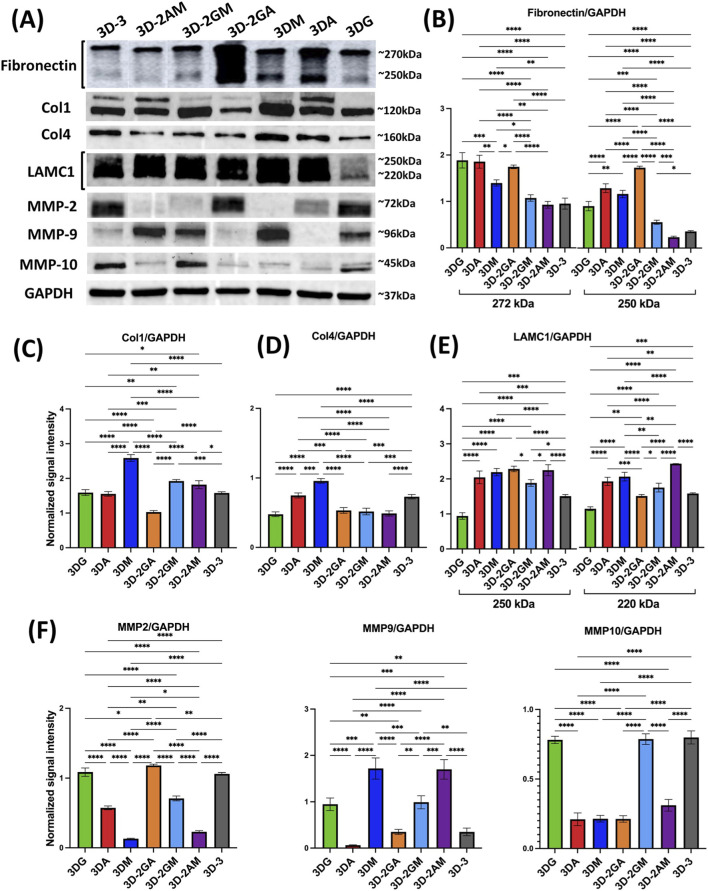
Expression of extracellular matrix proteins in homotypic and heterotypic 3D brain tumor models. **(A)** The immunoblotting analyses of ECM proteins in spheroids. One representative Western blot of two independent experiments is shown. The quantification of the protein expression was normalized to GAPDH as a loading control. The protein bands of target were quantified as relative values to loading control bands and the normalized signal intensity results are shown in the graphs for the following ECM proteins: **(B)** Fibronectin, **(C)** Col1, **(D)** Col4, **(E)** LAMC1, **(F)** MMP2, MMP9, MMP10.

Fibronectin functions as a substrate for tumor cell adhesion and invasion, thereby facilitating the infiltrative growth of gliomas (Wei et al., 2024). The analysis yielded two isoforms with molecular weights of 270 and 250 kDa, as illustrated in Figures 8A,B. Among the cellular models examined, elevated levels of fibronectin isoform synthesis were observed in homotypic spheroid models, with a subsequent decrease in heterotypic models, with the exception of 3D-2GA. Increased fibronectin synthesis in the 3D-2GA model reproduces features similar to astrogliosis, defined as astrocyte activation in the presence of glioma cells (Wu et al., 2025). These data are consistent with recent studies showing that activated astrocytes, by depositing fibronectin into the extracellular matrix, can further maintain their pro-inflammatory phenotype in an autocrine manner (Chu et al., 2023). Furthermore, our observation of increased fibronectin expression correlates with *in vivo* data, where reactive astrocytes serve as the primary source, synthesizing and depositing fibronectin fibrils (Werkman et al., 2020). Thus, the developed 3D model allows us to reproduce individual features progressive tumors.

Type I collagen (Col1) has been demonstrated to promote cell invasion and enhance tumor formation via the PI3K/Akt signaling pathway (Wang et al., 2022). A detailed analysis of Col1 synthesis revealed a pivotal role for microglia. The level of the substance was found to be at its zenith in 3DM (Figures 8A,C). In the context of formed heterotypic spheroids, which also contained microglia, there was a notable enhancement in collagen synthesis when compared to homotypic models. However, an exception was observed in the 3D-2GA model, where Col1 levels were found to be minimal. This finding suggests that microglia play a pivotal role in collagen remodeling in heterotypic models. Glioma cells have been observed to produce and remodel type IV collagen (Col4), thereby creating a supportive scaffold that facilitates their invasion (Guzdek et al., 2025). A thorough analysis of Col4 reveals that its expression varies among 3D models, with increasing levels observed in the following sequence: 3DM > 3DA > 3DG (Figures 8A,D). For the 3D-2, Col4 levels were comparable to those in the 3DG model. The 3D-3 model exhibited a marginal increase in Col4 levels in comparison to the 3D-2. The diminished expression of Col4 in heterotypic models may signify a shift in the equilibrium between distinct types of collagens in response to intercellular interactions, particularly with glial cells (G), in which the expression of this collagen is negligible. The potential for pro-inflammatory cytokines, such as IL-1β and TNF-α, released by activated microglia, to suppress collagen IV synthesis through the activation of metalloproteinases that degrade basement membranes is a plausible hypothesis.

The glioma tumor microenvironment has been found to secrete various laminin molecules, with subtypes of these molecules exerting specific effects on glioma cell invasion *in vitro* (Bai et al., 2024). The data on the expression of the laminin γ1 subunit (LAMC1) in various components of the glioma microenvironment, as presented in Figures 8A,E, demonstrate a specific pattern. The 3DG model is distinguished by its reduced LAMC1 content in comparison to the 3DA and 3DM. In heterotypic models containing astrocytes, the protein level was found to be highest; however, in the 3D-3 model, it was the lowest among the co-culture models. The data suggest that in the glioma model, the primary source of LAMC1 is not the tumor cells themselves, but rather the resident cells of the tumor stroma, primarily microglia and reactive astrocytes.

The subsequent phase involved the evaluation of the expression levels of metalloproteinases (MMPs), including MMP-2, MMP-9, and MMP-10, which play a pivotal role in the active degradation and remodeling of the ECM (Figures 8A,F). MMP-2 and MMP-9 are gelatinases that primarily degrade type IV collagen, the main structural component of the basement membrane (Aitchison et al., 2025). Among homotypic models, the level of MMP-2 exhibited a typical variation in the order 3DG > 3DA > 3DM. A high level of MMP-2 expression, comparable to that observed in 3DG, was observed in heterotypic models containing tumor cells but not astrocytes. Lower levels of MMP-2 expression were detected in 3DM and 3D-2AM, indicating that U87MG glioma cells, rather than microenvironmental cells, made the greatest contribution to the expression of this MMP in the model. Conversely, the reverse pattern was observed for MMP-9, where levels were elevated in 3DM and heterotypic models containing microglial cells and diminished in models containing tumor cells and astrocytes. MMP-10 functions as a stromelysin and a regulator of the activity of other types of MMP, modulating fibronectin and proteoglycans. A relatively high level of activated MMP-10 was observed in 3DG and 3D-2GM, as well as in the 3D-3. These findings corroborate data on MMP-10 expression by gliomas (Aitchison et al., 2025).

Consequently, the 3D-3 cell models mirror glioma progression, which is accompanied by active and cooperative synthesis of ECM proteins. The formation of a rigid collagen matrix is initiated by microglia, while reactive astrocytes supply fibronectin and laminin to organize invasion pathways. The data obtained on MMP activity in the model confirm that cells of the TME are also involved in the activation of the tumor’s aggressive potential. To verify the obtained data at the functional level, the invasive potential of cells was assessed in heterotypic spheroid models using matrices of varying stiffness, which allowed for the simulation of *in vivo* microenvironment conditions.

### 3.6 The invasive potential of cells in heterotypic spheroid 3D models of brain tumors

Glioblastoma cell invasion is a complex process involving various biological mechanisms, including intercellular adhesion, degradation of the ECM and associated adhesion molecules, and the molecular action of enzymes in the TME of glioblastoma and stromal cells (Doroszko et al., 2025; Xu et al., 2025). In experimental cell biology, the terms “migration” and “invasion” are clearly distinct from one another (Kramer et al., 2013). To emulate glioma “migration” and “invasion” processes *in vivo*, matrices of varying stiffness were utilized, including 2% gelatin and 50% Matrigel™.

**FIGURE 9:**
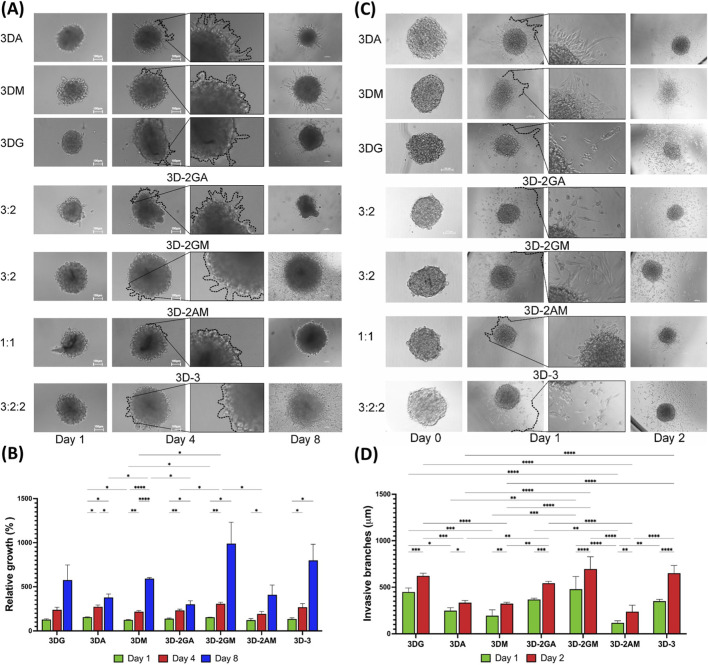
Analysis of the on-top invasive potential and migration capacity of homo- and heterotypic 3D spheroid models of brain tumors using Matrigel™ as a substrate. **(A)** Invasion of cells from 3D cellular models. Invasive cell outgrowths are indicated by a dashed line. Scale bar: 100 μm. **(B)** Graph representing the relative growth of glioma 3D cellular models on Matrigel™ substrate. **(C)** Representative images of cell migration from 3D spheroids. Dashed lines delineate migration branches. Scale bar: 100 μm. **(D)** Graph representing the maximum outgrowth distance of migration branches (mm) for each spheroid model. Data are presented as mean values (M) with standard deviation (SD), calculated from triplicate independent measurements. Differences between experimental groups were considered statistically significant at *p < 0.05; **p < 0.01; ***p < 0.001; ****p < 0.0001 (two-way ANOVA with Shapiro–Wilk test).

Tumor cells and astrocytes within 3D on a Matrigel™ substrate began to invade the surrounding environment by days 1–4 of culture, in contrast to microglia, those on-top invasion was activated only by day 8. It is noteworthy that by day 8, the tumor cells had invaded a greater distance, while the astrocytes and microglia had invaded in a similar manner, spreading along the periphery of the spheroid boundaries (Figure 9A). Consequently, diverse cell types within the spheroid manifest disparate rates of invasion activation, exhibiting either moderate activation in the early stages (days 1–4) or delayed activation (by day 8) on the Matrigel™ substrate. This phenomenon may be associated with the synthesis of various MMPs by tumor cells and microenvironmental cells (Figure 8F). In a similar manner, the 3D-2GA and AM demonstrated moderate invasion activation in the initial stages (days 1–4). Conversely, the 3D-2GM and 3D-3 exhibited active on-top invasion with a delay by day 8 (Figure 9B).

It has been observed that tumor cells exhibit a behavior similar to “gelatin phagocytosis,” in which they break down the gelatin matrix, indicating a high invasive potential (Björklund and Koivunen, 2005). This suggests that there may be a greater distance of cell exit from the spheroid. Therefore, the test analyzed not the area of invasion, but rather the furthest point of cell exit from the spheroid. Using a similar invasion assay on a gelatin substrate, it was shown that cells in 3DG exhibited high invasive potential compared to cells in 3DA or 3DM (Figures 9C,D). It has been observed that heterotypic spheroid models may exhibit increased cell invasion in comparison to homotypic models. The 3D-2GA was an exception, showing a lower invasive potential on a gelatin substrate compared to astrocytes or microglia cultured in 3D. The results obtained on a gelatin substrate suggest a potential role for MMP-2 and MMP-9, as gelatin is considered the primary substrate for gelatinases.

Therefore, the results suggest that the temporal dynamics and spatial pattern of invasion in the TME are influenced by the properties of tumor cells and the specific contribution of various types of stromal cells (astrocytes and microglia). Each of these features is characterized by a unique MMP expression profile.

### 3.7 Response spheroid to temozolomide treatment

**FIGURE 10:**
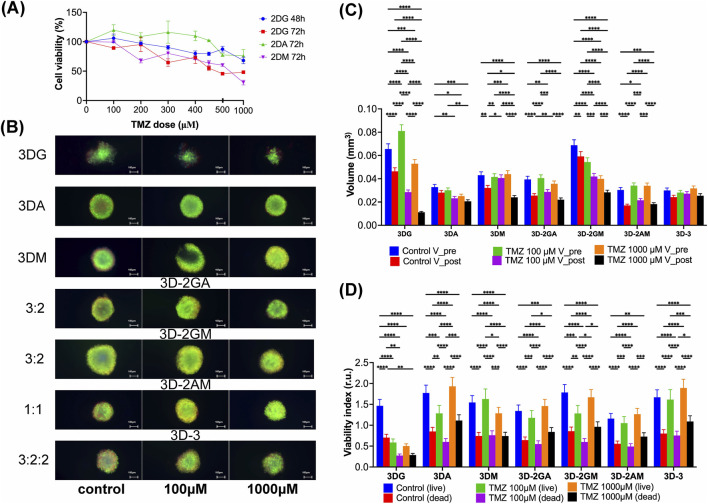
Characterization of homo- and heterotypic spheroid of glioma to treatment of TMZ. **(A)** Cells were treated with increasing concentrations of TMZ (0–1000 µM) for 48-h, and 72-h. MTT assay was performed to detect cell viability and IC50 (the half-maximal inhibitory concentration) values. **(B)** Live/Dead fluorescence microscopy images of spheroids after drug exposure (green, live; red, dead; blue, total nuclei of cells). Scale bar: 100 μm. **(C)** Changes in spheroid volumes during the sample preparation stage for live/dead staining. V_pre - before the dyeing process; V_post - after the dyeing processes. **(D)** A comprehensive analysis of alterations in signal intensity was conducted from both living and deceased cells. The calculations were meticulously executed on a total number of cells, with the fold change in the volume of the spheroid during the sample preparation phase being a crucial factor in the analysis. Data are presented as mean values (M) with standard deviation (SD), calculated from triplets of independent measurements. The difference between the experimental groups was statistically significant at *p < 0.05; **p < 0.01; ***p < 0.001; ****p < 0.0001 (one-way ANOVA with Shapiro-Wilk test).

Temozolomide (TMZ) remains the standard-of-care chemotherapy for glioblastoma and aggressive IDH-mutant gliomas (Stupp et al., 2005). A comprehensive review of the extant literature was conducted, which informed the testing of concentrations of the drug TMZ ranging from 0 μM to 1000 μM and exposure times of 48 and 72 h on 2D models (Kriuchkovskaia et al., 2026; Poon et al., 2021; Vasileva et al., 2024). Following a 48-h of exposure to TMZ in a 2D model of U87MG tumor cells (IC50 = 1473 μM), the cells demonstrated viability within the selected concentration range. This outcome is in contrast to the results observed after 72 h of exposure (IC50 = 478 μM) (Figure 10A). It is worthy of note that, in contrast to microglia (HMC3) (IC50 = 673μM), astrocytes (CCF-STTG1) (IC50 = 1879 μM) exhibited resistance to the chemotherapy drug TMZ across the full range of concentrations tested after 72 h in 2D (Figure 10A). In order to further analyze the data, doses of 100 μM and 1000 μM were selected for the spheroid models.

The Live/Dead staining revealed a decrease in the number of live cells (green signal) in 3DG (Figure 10B). However, it should also be noted that the size of the spheroid decreased in the analysis. For other homo- or heterotypic models, visual analysis did not permit a clear assessment of changes in the ratio of Live/Dead cells; however, changes in spheroid size were also observable. The subsequent stage of the analysis therefore involved the evaluation of alterations in spheroid volume (Figure 10C) and the calculation of the mean integrated density of FDA+ (live) or PI+ (dead) to Hoechst 33342+ (total) cells across the models (Figure 10D).

A thorough analysis of the spheroid volume, both prior to and following the staining procedures, revealed a significant decrease in volume. This decline may be indicative of a decline in cell viability and the loss of dead cells during sample preparation (Figure 10C). The alterations in the volumes of spheroids on the 0th and 3rd days of experimentation have been illustrated in Supplementary Figure S6A.The most significant decrease in volume was observed in homo- (3DG and 3DM) and heterotypic (3D-2GA, 3D-2GM, and 3D-2AM) spheroid models, and was more pronounced at a TMZ concentration of 1000 μM. A comparative analysis of the mean integrated density (live or dead) was conducted, with the ratio of changes in the volume of spheroids (k-parameter) being taken into account (viability index). This analysis revealed the varying sensitivities of the spheroid models to TMZ. The most marked alteration in viability index was observed in the 3DG model, with the 3DA model demonstrating resistance to the action of the drug (Figure 10D). Heterotypical models are distinguished by the occurrence of resistance to TMZ action at the concentrations under investigation. Moreover, an increase in the proportion of dead cells is observed at a concentration of 1000 μM.

A comprehensive analysis of the MTT test at 48 and 72 h was conducted, revealing a consistent pattern across several models (Supplementary Figure S6B). However, it was observed that certain models exhibited an opposite response, characterized by the activation of cell proliferation in response to the drug. This finding has the potential to result in the interpretation of a false result. It has been established that TMZ functions by acting on intercellular connections between cells, thus disrupting them (Jiang and Penuela, 2016; Schmidt et al., 2025). This process subsequently facilitates the penetration of the MTT reagent into the spheroid and the subsequent release of formazan crystals during analysis (Ghasemi et al., 2021). This, in turn, leads to an increase in signal detection and can be interpreted as cell proliferation.

Consequently, it can be posited that the TME cells can exert a substantial influence on the pharmacodynamics of drugs. In the development and analysis of new therapeutic approaches, it is essential to consider both the three-dimensional structure *in vivo* and the use of more complex cellular models that incorporate tumor-associated cells.

## 4 Discussion

Spatial mapping shows that gliomas consist of both disorganized and structured regions. Structured regions display a five-layered organization that is associated with hypoxia and extends beyond the organization that is visible by histology (Greenwald et al., 2024). Accounting for heterogeneity is clinically significant; it also creates challenges in studying complex intercellular interactions and the effects of individual cells in a controlled manner. This is due to the unknown number and ratio of cells, as well as unknown cellular phenotypes (Doroszko et al., 2025; Jang and Park, 2025; Zheng et al., 2025). The study of spheroid growth and formation confirms the physiological relevance and significant role of microenvironmental cells in influencing the growth dynamics and compaction of the model. It was observed that the dynamics of spheroid volume increase were negatively affected by the number of astrocytes in 3D-2 and 3D-3, due to the promotion of spheroid compaction by astrocytes (Figures 1, 2; Supplementary Figure S1). Astrocytes are characterized by their high protein content, which is instrumental in regulating their adhesion properties. This is exemplified by the presence of cadherins, laminin, and integrins, which play a crucial role in the formation of a compact glioma spheroid (Efthymiou et al., 2021). Another factor that may enhance spheroid compaction is the ability of glioma cells to induce the transition of astrocytes into a reactive state. Although GFAP levels did not significantly change in heterotypic conditions compared to astrocyte monoculture (Figure 7A), which precludes using GFAP alone as a marker for astrogliosis in 3D model, other features commonly associated with astrocyte activation were detected. The observed upregulation of fibronectin and changes in SNAIL expression recapitulate certain astrogliosis-like features, in line with the emerging concept that reactive astrocytes can exhibit context-dependent molecular signatures (Wu et al., 2025). Thus, we are able to state that the developed 3D-2GA model reproduced some distinctive signs of astrogliosis. As previously described by Heinrich et al. (2024), such an increase in GFAP levels in a heterotypic model was associated with a compaction effect (Heinrich et al., 2024). Furthermore, we were able to demonstrate a similar effect of microglia on the compaction of 3D models, observing a reduced volume in spheroids with a high microglia ratio (Figures 1, 2; Supplementary Figure S1).

Recent studies employing contemporary omics methodologies have demonstrated that the structural organization of glioblastoma exhibits a tendency towards either a highly structured or a loosely structured configuration. This phenomenon appears to be contingent upon the unique characteristics inherent within the individual tumor (Ruiz-Moreno et al., 2025; Zheng Y. et al., 2026). Glioma cells are found in spatially segregated regions, termed “niches”, which form complex ecosystems with microenvironmental cells, including neurons, glia, blood vessels and immune cells. These ecosystems support glioma cell growth and treatment resistance (Ren et al., 2023). In spheroids composed of multiple cell types, minimizing the energy associated with surface tension leads to “phase separation” (Shafiee et al., 2019) – a phenomenon in which cells sort themselves by homotypic adhesion and form distinct cell clusters (Abedin et al., 2023). The spatial configuration of cells in a spheroid composed of tumor cells and microglia cells has recently been delineated (Garcia-Saez et al., 2026). Over the course of a week, the spheroid underwent growth, forming a distinct “core-shell” structure. The glioma cells were concentrated in the central part (core), while the periphery was occupied by a layer of microglia. The present study’s analysis of the relative positioning of cells at the onset of spheroid formation aligns with the findings reported by Garcia-Saez et al. (2026) concerning the formation of a “core-shell” structure (Garcia-Saez et al., 2026). Nevertheless, the RT analysis of the spheroid structure (1–48 h) unveiled novel spatiotemporal patterns, characterized by the formation of two distinct poles (Figures 4, 5). The highest degree of cell polarization was achieved in the 3D-3, characterized by the formation of astrocyte microclusters and separated poles consisting of microglia and glioma cells. It is important to acknowledge that the differential adhesion hypothesis, proposed by Malcolm Steinberg, is the primary explanation for this phenomenon (Foty and Steinberg, 2025). This hypothesis posits that cells are sorted based on differences in tissue surface tension. Cells that exhibit higher levels of intercellular adhesion (e.g., those with higher cadherin expression) form stronger and more stable contacts. Consequently, they actively migrate toward the inner core, while less cohesive cells surround them in the outer layer. Secondly, in addition to adhesion, the generation of cortical tension by the actomyosin cytoskeleton is a factor that causes cells to round up and minimize contact with neighboring cells (Mehes et al., 2023). Discrepancies in contractile capacity can precipitate rapid cell separation over brief time periods, thus superseding adhesion during protracted periods. Diep et al. (2023) demonstrated in a heterotopic spheroid model that microglia gradually accumulated at the glioblastoma boundary and penetrated into the glioblastoma region, forming a glioblastoma-microglia assembloid (Diep et al., 2023). This phenomenon was analogous to that observed in a xenograft model and in tissues from patients with glioblastoma (Ricard et al., 2016). It is important to note that in other organoid models, microglia also demonstrate the ability to form distinct clusters rather than distribute themselves uniformly (Ali et al., 2022). Astrocytes also accumulated at the interface between the glioblastoma mass and microglia, forming an astrocytic scar barrier (Diep et al., 2023). In the model under consideration, the position of the astrocytes is not altered; rather, the astrocytes form a dense central core around which the glioma cells organize themselves (Figures 4, 5). Cui et al. (2023) utilized 3D hydrogels to investigate the interaction between glioblastoma and astrocytes, thereby demonstrating cell-specific disparities in the migratory behavior of U87MG and LN229 cells in the presence of astrocytes (Cui et al., 2023). Moreover, the research undertaken by Briot et al. and Heinrich et al. demonstrates that the presence of astrocytes (in conjunction with microglia) has a considerable impact on the response of gliomas to therapy (Briot et al., 2025; Heinrich et al., 2024). These findings underscore the high plasticity of the TME and the critical role of microenvironmental cells in the spatial organization of a heterogeneous tumor, a phenomenon that is not captured by endpoint analysis. It is imperative to underscore the significance of modeling the tumor margin enriched with microglia for the study of several aspects of tumor progression and drug resistance (Briot et al., 2025). Thus, microglia cells, located at the border of the invasive front, create a “border niche” and form onco-streams, facilitating the invasion of tumor cells (Kang et al., 2025). Furthermore, the conditions of the border niche are characterized by an enrichment of CSCs that activate microglia with an M2 (anti-inflammatory) phenotype, facilitating tumor growth and invasive processes (Bryukhovetskiy et al., 2016). Briot et al. (2025) demonstrated that glioma cells rapidly displaced astrocyte and microglia populations, reflecting a strong capacity for regeneration following early intercellular competition and cell death (Briot et al., 2025). This may also provide a theoretical framework to explain the formation of distinct cell clusters in the model under investigation. It has been observed that microglia predominate at the invasive front, whilst clusters of monocytic macrophages are present in perivascular and necrotic zones (Cheng et al., 2026).

Spheroids are distinguished by their capacity to generate dense and compact structures, which contributes to the formation of a necrotic core (Abdurakhmanova et al., 2025). All examined spheroids exhibited high cell viability (Figure 3A). However, analysis of intracellular ATP revealed differences depending on cellular composition. The 3DG exhibited maximum ATP levels, indicative of their aggressive, proliferative phenotype (Figure 3B). The incorporation of microenvironmental cells (astrocytes and microglia) into the spheroid composition resulted in a reduction of the total ATP pool. The elevated ATP levels observed in the 3DG are consistent with the concept of the Warburg effect. This phenomenon can be attributed to the inefficient pathway of anaerobic tissue respiration, which is compensated by an increased energy consumption necessary to maintain high proliferative activity (Strickland and Stoll, 2017; Comelli et al., 2018). The voltage-dependent dye JC-1 was utilized to evaluate mitochondrial functionality. The findings of this study indicate that the presence of reduced JC-1 levels in glioma cells is indicative of mitochondrial depolarization (Supplementary Figure S2). The higher rate of ATP production under glycolytic conditions compared to the oxidative phosphorylation cycle leads to ATP accumulation (Martínez-Reyes et al., 2020). The low ATP levels in 3DA reflect a relatively low proliferation rate, as less ATP is required for a growing tumor (Baharuddin and Yusoff, 2025). The reduced availability of ATP has been shown to trigger the activation of AMPK, which in turn causes astrocytes to shift from normal metabolism to alternative energy sources (Wu et al., 2025). In the context of 3DA, the lowest ATP level has been shown to correlate with the basal state of astrocytes in monoculture. ATP is a pivotal signaling molecule that facilitates communication between astrocytes and microglia under conditions that closely resemble *in vivo* settings (Shinozaki et al., 2014). A tendency towards increased ATP levels was identified in 3D-2AM in contrast to the levels observed in 3DA, or alternatively, a decline relative to the 3DM levels. It can be hypothesized that this phenomenon is associated with the activation of astrocytes by pro-inflammatory cytokines from microglia, which induces a transition to a reactive state with increased energy requirements (Liddelow et al., 2017). Consequently, astrocytes have the capacity to secrete regulatory factors that maintain microglia in a homeostatic state (Gavillet et al., 2008). The mean ATP level in 3DM (Figure 3B) seems to mirror the energy expenditures associated with immune surveillance (Wu et al., 2024). In contrast to tumor cells, astrocytes and microglia within heterotypic spheroids exhibited higher levels of JC-1. This finding correlates with high mitochondrial membrane potential and the capacity for oxidative phosphorylation to maintain energy balance (Supplementary Figure S2). The presence of astrocytes has been demonstrated to modify the interaction between glioma and microglia. One hypothesis suggests that astrocytes may function as a metabolic buffer, thereby facilitating the redistribution of energy resources among cells within the microenvironment (Matias et al., 2018; Park et al., 2025). Alternatively, astrocytes activated by glioma signals may secrete factors that shift microglia toward a more tolerant phenotype, thereby attenuating their suppressive effect on tumor cells.

In homotypic spheroids, the co-expression of cytokines (GRO-α, IL-8, IL-6) was observed (Supplementary Figure S7), suggesting the presence of shared transcriptional regulation by NF-κB or AP-1 (Ramar et al., 2025; Shah and Lucke-Wold, 2025). The induction of these cytokines is typically observed during the process of innate immune activation, and their actions are often synergistic. In homotypic models, the presence of two antagonistic clusters (pro-inflammatory and neurotrophic) was detected, suggesting the existence of an internal “molecular switch” that maintains a balance between two distinct functional programs. The transition to heterotypic models has been shown to disrupt this structure. The disintegration of the GRO-α/IL-8/IL-6 cluster signifies that cytokine production becomes independently regulated via paracrine signals. Astrocytes have been observed to suppress IL-6 production by glioma and microglia, thereby disrupting initial co-regulation (Cui et al., 2026). The most notable findings were synergism in 3D-2AM (IP-10 secretion) and 3D-2MG (MIP-1α secretion). The exclusive increase in IP-10 in models containing astrocytes and microglia (3D-2AM and 3D-3) suggests a mechanism for T-cell recruitment, which could indicate an attempt to stimulate anti-tumor immunity. It is noteworthy that glioma cells in 3D-3 did not fully suppress this mechanism, thereby providing a potential “window of opportunity” for immunotherapy (Xu et al., 2023; Shu et al., 2026). The activation of TNF signaling in stromal cells has been shown to induce IP-10 expression, leading to an increase in T-cell infiltration and the achievement of remission in models of resistant glioma (Du et al., 2025). The augmented secretion of MIP-1α (CCL3) in 3D-2GM, concomitant with its attenuation in the presence of astrocytes in 3D-3, underscores the distinct nature of the tumor-microglia interactions in our model. In light of the well-established role of MIP-1α as a chemoattractant for myeloid cells, its overproduction by microglia could be expected to promote myeloid recruitment. However, this effect may be counterbalanced by astrocytes, suggesting a modulatory function. A regulatory role for astrocytes in the glioma microenvironment has been previously documented. For instance, astrocytes have been shown to abolish microglia- and macrophage-facilitated glioma cell death induced by Smac-mimetics (Malone et al., 2024). Astrocytes play a dual role. In concert with microglia, these cells induce IP-10 production, while concurrently repressing pro-inflammatory cytokines IL-6 and MCP-1. A reduction in IL-6 levels in the presence of astrocytes (3D-2GA, 3D-2AM, 3D-3) has been shown to offer protection against a cytokine storm (Li Y. F. et al., 2025). However, in the context of glioma, this may promote tumor progression by establishing an environment that is less inflammatory and more conducive to growth. Astrocytes and glioma cells form a STAT3/IL-6-mediated feedback loop, and decreased IL-6 in the presence of astrocytes may indicate disruption of this loop or reflect astrocytes’ “guardian” function of suppressing inflammation unless over-activated by the tumor (Li Y. F. et al., 2025). The 3D-3 model exhibited a unique profile, not simply the sum of paired cultures (e.g., for MCP-1 or MIF), underscoring the need for complex *in vitro* models that resemble *in vivo* conditions for therapeutic screening. Heterotypic 3D models comprising astrocytes and microglia exhibit key microenvironmental interactions and display properties absent in homotypic spheroids, including the formation of a dense stromal barrier and the induction of immunosuppressive genes (Heinrich et al., 2024). The employment of microfluidic models, comprising endothelial cells, astrocytes, and glioma stem cells, has facilitated the discernment of distinct ligand-receptor interactions (e.g., SAA1-FPR1) implicated in chemotactic invasion, a phenomenon that eludes detection in simplified monocultures (Adjei-Sowah et al., 2022). New strong correlations in heterotypic cultures (e.g., MCP-1/LIF, HGF/SDF-1α) reflect the formation of functional connections at the microenvironmental level (see Supplementary Figure S4). The MCP-1/LIF correlation is presumably attributable to the fact that both are produced by astrocytes and microglia in response to mutual stimulation. LIF has been demonstrated to maintain tumor cell stemness (Penuelas et al., 2009), while MCP-1 has been shown to recruit tumor-associated macrophages (Hambardzumyan et al., 2016). The co-regulation of these genes may provide a molecular basis for the association between macrophage infiltration and stem cell phenotype. The positive correlation between HGF and SDF-1α in heterotypic cultures is of particular interest, as both are key mediators of glioma invasion (Xia et al., 2025; El Kheir et al., 2024). Thus, the heterotypic glioma microenvironment induces a qualitative reorganization of the regulatory cytokine network. The implementation of correlation analysis and hierarchical clustering techniques yielded the identification of a breakdown of intracellular co-regulatory modules and the emergence of novel activation links. Glioma cells, astrocytes, and microglia in co-culture function not as the sum of isolated elements, but as a new system with its own regulatory logic.

In the context of glioma, there are at least three specialized tumor niches that include the vascular network as an integral regulatory component: the perivascular tumor niche, the vascular-invasive tumor niche, and the hypoxic-necrotic tumor niche. The functionality of these niches is contingent not only on the presence of normal cellular components within the tumor microenvironment, but also on the genetic and epigenetic profiles of CSCs (Alves et al., 2021; Lee et al., 2025). Spheroids are characterized by an absence of a vascular network, resulting in inadequate oxygen and nutrient supplies (Beckers et al., 2025). In particular, cells in the central region of the cluster experience a higher level of stress compared to cells in the outer layer of the spheroid. Consequently, cells within the spheroid are exposed to low nutrient levels and hypoxia. A thorough examination of CSC markers reveals that the 3DA and 3DG models exhibit high expression levels of CD133, CD44, and CD24. In contrast, the 3DM model is distinguished by a high proportion of CD44^+^-cells (Figure 7A). This finding aligns with previously published data on the abundance of CSC subpopulations within the glioma structure (Alves et al., 2021). Cells located at the periphery of a hypoxic tumor, along with a distinctive morphological characteristic known as pseudo-palisading, have been observed to exhibit a reduced rate of proliferation and an increased propensity to engage invasive programs, thereby facilitating egress from the hypoxic core (Brat et al., 2004). As demonstrated by Brown et al., the manifestation of phenotypic changes is contingent upon microenvironmental conditions, thereby underscoring the capacity of CD133^+^-cells to undergo transformation into CD44^+^-cells under hypoxic conditions (Brown et al., 2017). Heterotypic models were distinguished by elevated expression of CSC markers in the presence of microenvironmental cells, thereby substantiating the established function of the glioma microenvironment in sustaining an invasive, aggressive population of CSCs (Fidoamore et al., 2016). A recent study has demonstrated that glioblastoma cells interact with CD24 via Siglec-10 and can modulate the TME by enhancing antitumor immunity, a process that contributes to immune evasion, thereby promoting an immunosuppressive environment (Kopecky et al., 2026).

For an extended period, it was widely accepted that HIF-1β (ARNT) is constitutively expressed. Subsequent studies have demonstrated that the expression of ARNT can be modulated in tumor cells in response to hypoxic conditions (Mandl and Depping, 2014). Furthermore, ARNT has been observed to form heterodimers not only with HIF-1α but also with other factors, such as AhR (aromatic hydrocarbon receptor), which has been shown to confer tissue-specific regulatory functions upon it (Ebersole et al., 2018). The ARNT protein synthesis exhibited no significant disparities among the examined models, whether in homotypic or heterotypic spheroid configurations (Figures 7B,C). However, the levels of protein synthesis and secreted VEGF in these models differed significantly (Figures 6, 7B,D). Upon initial observation, this finding appears to be at odds with the established function of the HIF-1 complex in the induction of VEGF. VEGF can be induced not only via HIF-1 but also via alternative signaling pathways (NF-κB, MAPK, PI3K/Akt), which are activated differently in various cell types. For instance, in microglia, VEGF is frequently induced via Toll-like receptor-dependent mechanisms that do not require HIF-1α (Bai et al., 2025; Li and Graeber, 2012; Richard, 2022). In glioma cells, oncogenic drivers (EGFR, PI3K) have been observed to maintain elevated levels of VEGF even under conditions of moderate hypoxia, a phenomenon that may not be directly associated with overall ARNT levels (Bonavia et al., 2012; Nicolas et al., 2019). Furthermore, Terashima et al. (2016) demonstrated that ARNT regulates VEGF expression in spheroids, but they used ARNT knockout systems, which highlights its necessity but not its proportionality (Terashima et al., 2016). Conversely, studies have demonstrated that CD133^+^-CSCs are the origin of the primary etiological factor driving neoangiogenesis in the tumor, VEGF (Gilbertson and Rich, 2007). The findings are consistent with prior research indicating the presence of a CD133^+^-CSC population within 3D glioma models (Figure 7A). These results are also in alignment with the documented high levels of VEGF expression exhibited by glioma cells in both homo- and heterotypic models (Figures 6, 7B,D). A notable observation is the decrease in VEGF expression levels within the 3D-3, suggesting adaptive reprogramming of the tumor in the presence of microenvironmental cells, with a reduction in the aggressive angiogenic phenotype and a shift toward other survival strategies.

The occurrence of the EMT in malignant gliomas remains a subject of debate, primarily due to the absence of typical epithelial characteristics in glioma cells (Xia, 2025). In contrast to epithelial tumors, glioma cells are characterized by their mesenchymal phenotype, which is attributed to their origin from the neuroectoderm. Consequently, the transition from E-cadherin to N-cadherin is not observed in these cells. In contrast, the EMT in glioma is characterized by increased expression of N-cadherin, vimentin, and specific transcription factors such as ZEB1/2, TWIST1/2, and SNAIL1/2 (Xing et al., 2022). A similar pattern of TWIST1 and SNAIL1/2 in various models indicates the coordinated activation of the EMT genetic program under the influence of specific microenvironmental signals. This ultimately aims to reduce intercellular adhesion and enhance the invasive potential of cells (Figures 7B,E–G). Vimentin was predominantly expressed by tumor cells and astrocytes, indicating that the mesenchymal signal in heterotypic models is primarily determined by the neoplastic and reactive glial compartments (Figures 7B,H). A study by Heiland et al. (2019) demonstrated that reactive astrocytes exhibit a distinct transcriptional phenotype associated with activation of the JAK/STAT pathway and release of anti-inflammatory cytokines. TGF-β, IL-10, and G-CSF (Heiland et al., 2019). Astroglia may be characterized by increased expression of EMT regulators in astrocytes, which enhances tumor invasiveness through microenvironmental remodeling (Gutmann, 2015). Conversely, reciprocal interaction between tumor cells and astrocytes enhances SNAIL activation in both populations, thereby simulating *in vivo* astrogliosis within the glioma microenvironment (Matias et al., 2018).

The TME, particularly the ECM, plays a pivotal role in glioma progression and therapy resistance. In spheroids, the secretion of VEGF by active cells can co-precipitate or form stable complexes with matrix proteins (e.g., fibronectin, collagen), thereby slowing its mobility and producing bands of 100 kDa and above (Figures 7B,D). This phenomenon is referred to as the “matrix-bound pool” of VEGF. Fibronectin is a multifaceted protein that exists in two distinct forms: soluble plasma and insoluble cellular. The latter is a significant ECM component that plays a crucial role in cell adhesion, growth, and migration. Subsequent analysis yielded the presence of two fibronectin isoforms at 270 and 250 kDa (Figures 8A,B). The 250-kDa form has been identified as the most prevalent monomer (Leonteva et al., 2026; Dalton and Lemmon, 2021), while the 270-kDa isoform is derived from alternative splicing (EDA/EDB domains). Elevated fibronectin levels were observed in homotypic spheroids, while heterotypic models exhibited a decline in fibronectin levels. The elevated levels of fibronectin observed in homotypic spheroids may be indicative of cellular stress and the subsequent attempt to generate an endogenous matrix for structural support. In heterotypic models, fibronectin decreased, possibly due to ECM remodeling driven by intercellular interactions. A salient finding was the elevated fibronectin levels observed in 3D-2GA compared to homotypic spheroids. This observation may be indicative of astrocyte triggering in response to glioma cells, as previously reported by Wu et al., in 2025. A recent study by Wang et al. (2022) revealed that collagen analysis indicated microglia as the predominant source of Col1 expression. This finding supports the hypothesis that glioma stem cell niches are sustained and that invasion plays a critical role in their development. Levels of Col1 were elevated in 3DM and increased in most heterotypic models, suggesting that microglia in co-culture adopt a pro-inflammatory, pro-fibrogenic phenotype, potentially driven by TGF-β secretion. In contrast, Col4 (a basement membrane component) exhibited high levels of expression in homotypic spheroids and repression in heterotypic cultures containing microglia or astrocytes. This phenomenon may be indicative of a shift in collagen balance, a process that is often influenced by pro-inflammatory cytokines derived from activated microglia. These microglia stimulate MMPs, which play a crucial role in the degradation of Col4. The expression of LAMC1, which has been shown to correlate with the malignancy of gliomas (Barbosa et al., 2024), was observed to be highest in models comprising astrocytes or microglia. Consequently, the primary source of this pro-invasive laminin component is not tumor cells themselves but rather infiltrating and reactive stromal cells. Overall, cell-cell interactions in the glioma model drive cooperative ECM remodeling: astrocytes form the fibronectin scaffold, while microglia supply Col1 and LAMC1 and simultaneously induce Col4 degradation, possibly via MMP activation. The binding of VEGF to the matrix, in conjunction with elevated expression of CSC markers in heterotypic models, establishes a stable niche that fosters invasion, angiogenesis, and the potential for therapy resistance.

MMPs promote tumor progression by degrading ECM components, releasing growth factors and chemokines, and facilitating angiogenesis and immune evasion (Aitchison et al., 2025; Zheng B. et al., 2026). Depending on spheroid composition, we observed different expression patterns of MMP-2, MMP-9, and MMP-10 (Figures 8A,F). MMP-2 and MMP-9 degrade Col4, a major basement membrane component, and their elevated expression in U87MG cells correlates with high invasiveness (Xue et al., 2017; Thanh et al., 2024). Notably, microglial cells in 3DM and 3D-2AM models showed elevated MMP-9 expression, consistent with published data showing that microglial MMP-9 supports glioma infiltration (Hu et al., 2014). Thorns et al. (2003) reported that MMP-2 and MMP-9 contribute to neoangiogenesis, while MMP-10 correlates with poor prognosis in astrocytic tumors. The elevated MMP-10 expression in the 3D-2GM model suggests that heterotypic interactions amplify diverse invasive factors.

Migration and invasion potential was assessed using two substrates: Matrigel™ (laminin, Col4, growth factors) and gelatin (denatured collagen). Matrigel™ evaluates barrier penetration, while gelatin specifically detects gelatinase activity (Passaniti et al., 2022; Derkach et al., 2021). Glioma cells invaded Matrigel™ within hours of contact (Abedin et al., 2023). Astrocyte spheroids showed early but localized on top invasion without long-distance spread. 3D-2GA and 3D-2AM exhibited moderate early invasion, whereas 3D-2GM and 3D-3 showed delayed but active late invasion (Figure 9). Astrocytic scar formation in Matrigel™ can limit tumor cell growth (Diep et al., 2023). Microglia remain quiescent until activated by microenvironmental or tumor-derived signals (Coniglio et al., 2016), explaining the delayed invasion. It is evident from the literature that microglia in contact with glioma cells also exhibit changes in cell morphology, transitioning from a branched to an amoeboid form (Resende et al., 2016). Friedel and Zou demonstrated a stage-dependent biphasic response of microglia to invasive glioblastoma cells. This response may be summarized as follows: an increased surveillance period occurs during sparse infiltration, followed by suppression of surveillance as the tumor burden increases (Friedel and Zou, 2026). In specific regions of the invasive front, notably peritumoral areas, microglia may establish “glial networks” around tumor infiltrates prior to complete mixing. There is evidence to suggest that microglia mobilize in advance of the invasive wave. This mobilization occurs in two phases: firstly, microglia envelop tumor infiltrates and, secondly, organize themselves into specialized “oncStreams” that direct collective migration (Kang et al., 2025). Glioma cells induce microglial MMP-9 expression via TLR-2/6 and p38 MAPK (Hu et al., 2014), and microglia-derived MMP-9 creates a proteolytic environment correlating with the late invasion (day 8) in microglia-containing models. The secretome of highly invasive models showed elevated LIF (stemness) and CCL3, which acts via CCR5; blocking CCR5 reduces invasion (Novak et al., 2020).

“Gelatin phagocytosis” is a mechanism by which invasive cells secrete proteases, phagocytose cleaved gelatin fragments, and degrade them intracellularly (Coopman et al., 1998). Gelatin migration assays (Figures 9C,D) showed that homotypic glioma spheroids exhibited the most distant outgrowth compared to astrocyte or microglia spheroids. Glioblastoma cells form invadopodia enriched in MMP-2 and MMP-9 to degrade ECM (Dinevska et al., 2025). Increased migration in heterotypic models (except 3D-2GA and 3D-2AM) correlated with altered MMP-2/MMP-9 expression (Figures 8A,F). Notably, MMP-9 in glioma tissue is primarily expressed by activated microglia/macrophages rather than tumor cells (Hu et al., 2014). For 3D-2GA and 3D-2AM, reduced migration on gelatin was associated with astrogliosis-like reactivity and formation of a tight barrier (e.g., fibronectin). Astrocytes secrete TIMP-1, which suppresses MMP-2/MMP-9 activity (Priego et al., 2025). FITC-Dextran penetration analysis (Supplementary Figure S8) confirmed the role of astrocytes in forming a physical barrier.

Temozolomide (TMZ) remains the primary chemotherapy drug for the treatment of gliomas; however, innate and acquired resistance mechanisms significantly limit its clinical efficacy (Pu et al., 2025). The principal factors contributing to the observed resistance to TMZ are DNA repair mechanisms, cell survival strategies and TME factors (Li H. et al., 2025a). It has been demonstrated that tumor cells and microenvironment cells vary in their sensitivity to TMZ, with alterations in the order of U87 MG > HMC3 > CCF-STTG1 (Figure 10A), which is consistent with previously described results (Herbener et al., 2020). In spheroid models, it has been observed that the microenvironmental cells present within the model significantly reduce its sensitivity to the action of TMZ, in contrast to the monotypic model of tumor cells (Figures 10B,D). Astrocytes and glioma cells communicate via gap junctions and exosome exchange to share proteins (such as ALKBH7), mRNA (such as MGMT mRNA) and upregulated channels (such as Connexin43) that facilitate the repair of DNA damage induced by TMZ in cancer cells (Gui et al., 2025; Yu et al., 2018) A further study demonstrated that astrocytes significantly reduced glioma cell apoptosis induced by the chemotherapeutic drugs TMZ and vincristine. The protective effect was found to be contingent upon direct contact between astrocytes and glioma cells via Cx43-GJC (Chen et al., 2015). Pustchi et al. (2020) demonstrated that in the co-culture, astrocytes increased GBM survival and resistance after combined drug treatment (TMZ+NF-κB inhibitor) compared to mono-cultures (Pustchi et al., 2020). A similar resistance to the action of the drug was also exhibited by microglia, in a manner analogous to that of astrocytes, in the spheroid model. Garcia-Saez et al. (2026) showed that heterotypic glioma-HMC3 spheroids exhibited increased proliferation and greater drug resistance to chemotherapy drug Temozolomide compared with homotypic spheroids. Furthermore, the results of this study suggest that glioblastoma resistance to TMZ can be enhanced by an interferon-driven gene expression profile induced by microglia, which in turn may have significant implications for the treatment of glioblastoma (Sorensen et al., 2024).

The data presented herein demonstrate that 3D spheroid models with heterotypic interactions are an adequate system for studying the cell-matrix and cell-cell interactions underlying the invasive phenotype of glioma, as well as for the search for therapeutic targets aimed at modulating the microenvironment. Nevertheless, it is imperative to acknowledge the limitations and controversial aspects inherent in our study.

## 5 Study limitations

The developed 3D spheroid models have several limitations. First, spheroids were generated using immortalized cell lines (U87MG, CCF-STTG1, HMC3), which may accumulate clinically irrelevant mutations during prolonged culture. Additionally, only one glioma line (U87MG) was used, which carries a homozygous PTEN mutation (Voloshin et al., 2023). The astrocytic origin of U87MG and CCF-STTG1 cells also complicates their discrimination in co-culture (Heinrich et al., 2024). It must also be noted that the cellular model under consideration consists of only three components, and it is not possible to fully replicate it *in vivo*. Future studies should incorporate primary cell cultures to better recapitulate tumor heterogeneity and improve translational relevance.

Second, the model does not include several TME components present in GBM, such as endothelial cells, pericytes, infiltrating immune cells, neurons, and oligodendrocytes (Chen et al., 2017). Incorporating endothelial cells would enable modeling of vascular-like structures and VEGF-mediated angiogenesis. The role of neurons and oligodendrocytes in supporting glioma growth, invasion, and drug resistance (Hide and Komohara, 2020) also remains to be addressed. *Ex vivo* brain slice models could complement spheroids by providing tissue-like architecture for studying invasion patterns.

Third, it is asserted that the utilization of transduced and labelled cells for the validation of migration and invasion will not enhance the reliability of the results. Conversely, this is likely to result in the production of additional artefacts and the generation of false signals for the following reasons: photobleaching and phototoxicity during prolonged observation, dye transfer between cells (via contacts and microvesicles), and direct effects on adhesion, proliferation, and migration (Lassailly et al., 2010; Vince et al., 1997; Zeng et al., 2016). Additionally, the intense signal from a dense spheroid can mask the weak signal from individual migrating cells (a process termed “masking”, “halo effect” and “background scattering”) (le Roux et al., 2008).

Fourth, the metabolic characterization was limited to ATP and mitochondrial potential measurements; quantitative assessment of glucose consumption and lactate production is needed. The influence of adhesion molecules (e.g., contactin) expressed by neurons and oligodendrocytes on glioma infiltration (Eckerich et al., 2006) also requires further investigation.

Fifth, it should be noted that the present study provides descriptive secretome profiling. Direct functional evidence for altered immune cell recruitment (e.g., Transwell™ migration assays with primary immune cells) was not obtained and represents an important direction for future investigation.

Sixthly, the dense packing of tumor cells in spheroids emulates the intercellular communication and interactions with the ECM that is characteristic of genuine brain tumors. This, in turn, complicates the subsequent adaptation of various assays designed to detect cellular toxicity and future interpretation. The employment of the classical MTT assay to evaluate cell viability within 3D spheroids may give rise to the production of misleading outcomes (Supplementary Figure S6B). In the case of a monolayer, where cells are accessible, the yellow MTT solution can penetrate its central layers quickly and evenly. By contrast, in a dense spheroid, this is not the case (Salata et al., 2025). The reaction product, insoluble violet formazan crystals, accumulates in the location where the reaction has occurred. In a spheroid, the local obstruction of further reagent penetration can result in uneven coloration. This approach consequently engenders a distorted representation of the spheroid’s viability, as opposed to an objective depiction. Furthermore, alterations to reagents during the course of the test have been shown to result in the dissolution of dead cells, thus giving rise to an erroneous interpretation. It is evident that alternative methods, such as WST-1, which forms soluble formazan, thus simplifying and accelerating the analysis process, or the luminescent ATP assay, which, despite its exceptional sensitivity, necessitates complete spheroid lysis, have the potential to generate more accurate and reliable results. However, metabolic dysfunction (e.g., for astrocytes) must also be considered. While this phenomenon can be explained within the framework of a monotypic model, heterotypic interactions can introduce distortions, thereby affecting the interpretation of data.

Finally, increasing spheroid complexity will inevitably increase the difficulty of culturing, sample preparation, analysis, and data interpretation.

## 6 Conclusion

Our model demonstrates one of the biologically relevant patterns, and its value lies in the ability to study paracrine interactions under controlled conditions. The pattern of protein-protein interactions reveals a highly integrated system in which extracellular matrix remodeling, cell motility, angiogenesis, and cell differentiation are tightly intertwined through common regulatory nodes, including MMP, chemokines, and transcription factors (Supplementary Figure S9). This organization enables cells to promptly respond to external signals and orchestrate such a sophisticated program as glioblastoma tumor progression. The study demonstrates that the developed 3D heterotypic models possess significantly higher physiological relevance compared to traditional homotypic or monolayer cultures. These cells effectively reproduce the complex, cooperative, and cell-specific ECM remodeling characteristic of the invasive margin of glioma. The created platform reflects the dynamic interactions leading to the formation of a tumor-supporting matrix (collagens, fibronectin, laminin) and the expression of MMPs. The presented models can serve as a powerful tool for further studying the molecular mechanisms of glioma invasion, searching for new therapeutic targets that disrupt these cooperative interactions, and screening drugs under conditions as close as possible to the physiological TME. A key direction for future research is the development of 3D spheroid models whose molecular and functional characteristics closely replicate the corresponding profiles of patient tumor tissues, thereby providing robust validation of their clinical relevance.

## Data Availability

The original contributions presented in the study are included in the article/Supplementary Material, further inquiries can be directed to the corresponding author.
