# Supplementary material for: Reconstructing the glioblastoma microenvironment in heterotypic 3D spheroids: a multicellular model to study tumor-stromal crosstalk
Source: Front Bioeng Biotechnol. 2026 Jul 3;14:1852454. doi: 10.3389/fbioe.2026.1852454 (PMC13376141; doi:10.3389/fbioe.2026.1852454)
Supplement: Supplementary file 1 [file DataSheet1.docx]

Supplementary Material

**
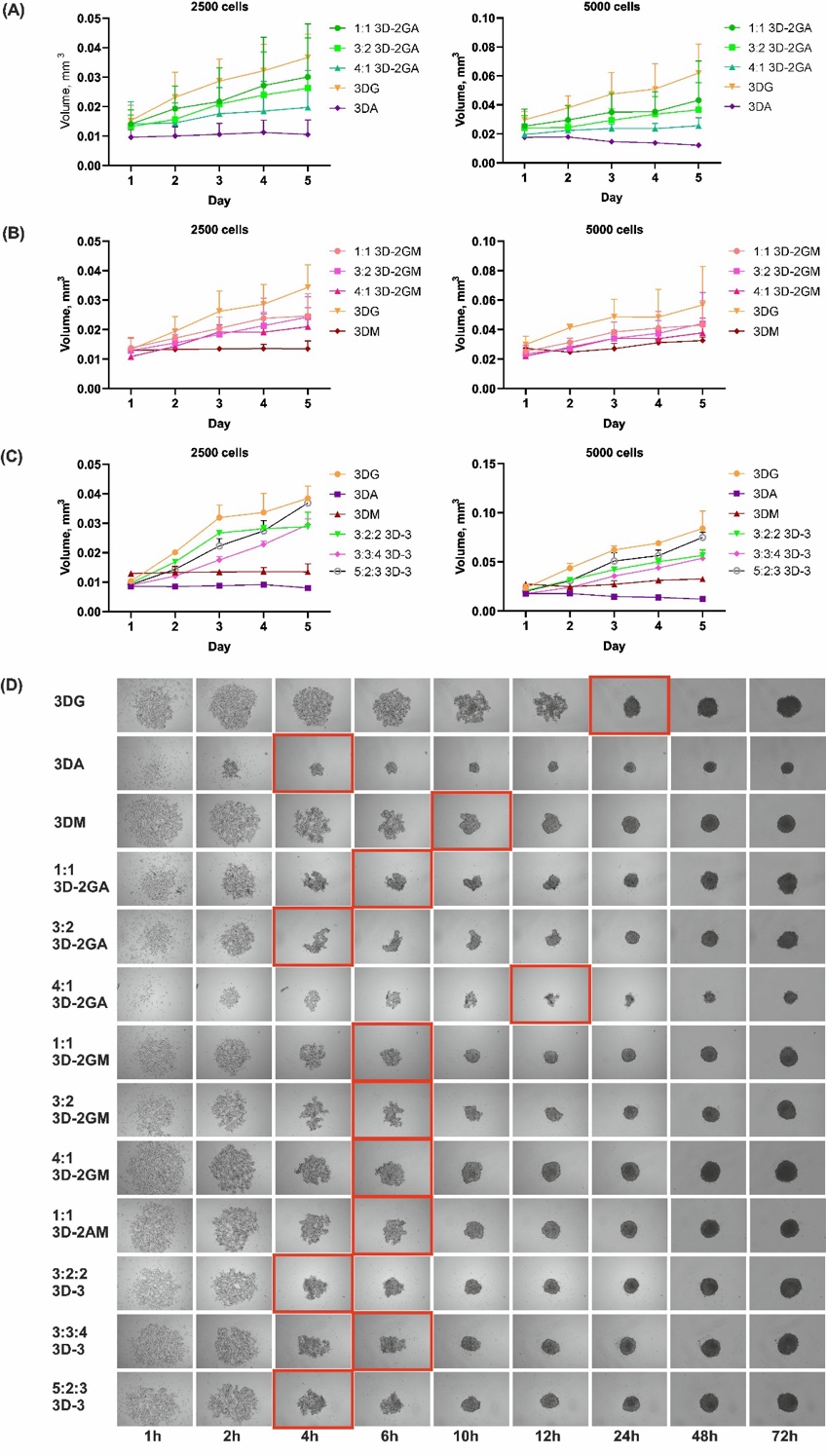
**

**Supplementary Figure S1.**  **The process of homotypic and heterotypic spheroid formation simulates different models of brain tumors depending on cell concentration and cellular composition of the 3D model.** Time-lapse photography of the 3D, 3D-2 and 3D-3 models. The data show **(A,B,C)** dynamics of changes in spheroid model volume depending on cell concentration and cellular composition of the 3D model. **(D)** Time-lapse photography of the 3D, 3D-2 and 3D-3 models. The micrographs show stages and times of spheroid formation.

**
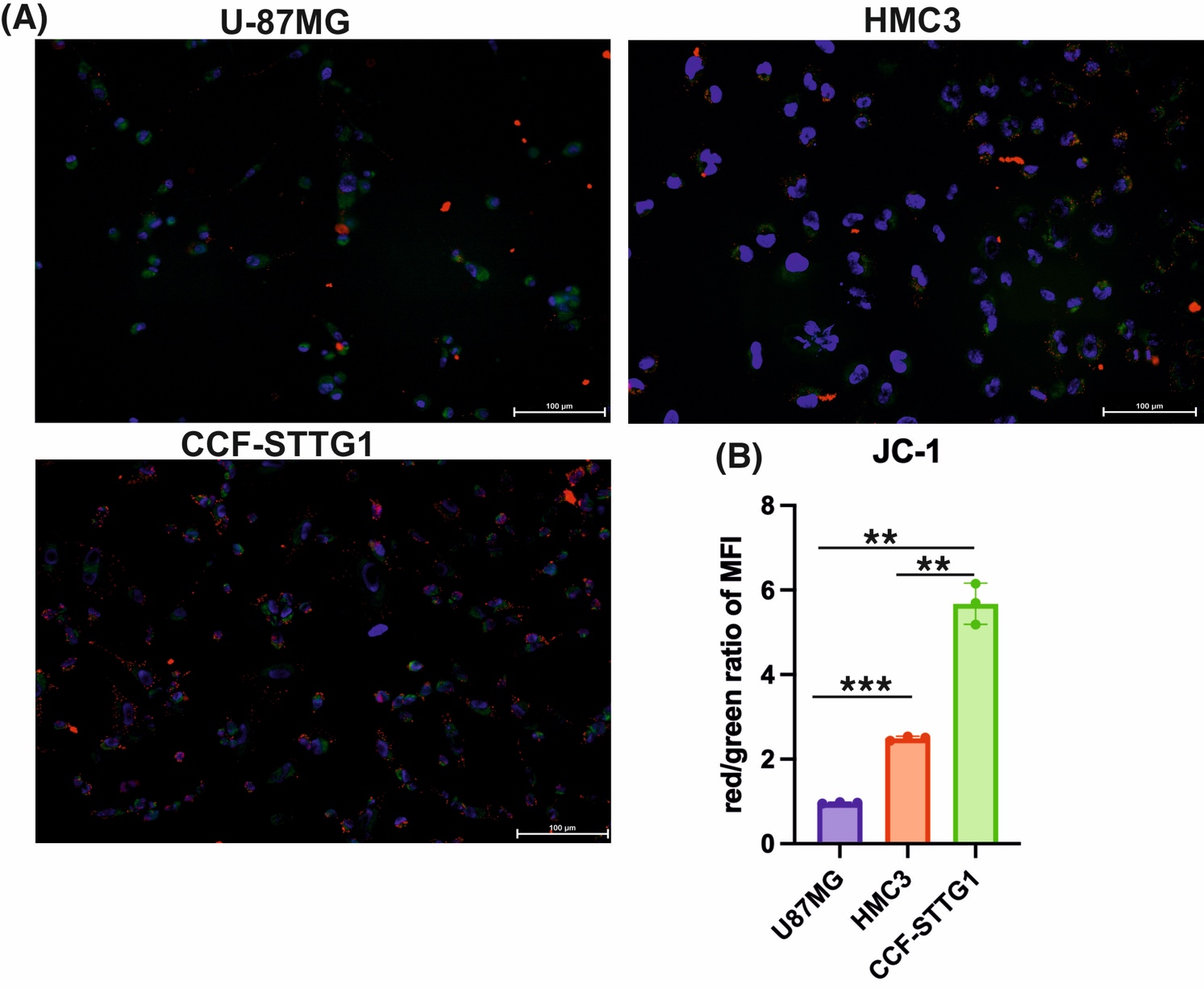
**

**Supplementary Figure S2.** **The evaluation of mitochondrial depolarization in U87MG glioma cells, HMC3 microglia, and CCF-STTG1 astrocytoma cells conducted through the utilization of fluorescence microscopy, employing the LumiTracker Mito JC-1 dye.** **(A)** The detection of JC-1 accumulation in healthy cells with normal ΔΨM in polarized mitochondria accompanied by the formation of red fluorescent J-aggregates and green fluorescent native JC-1 in cells with depolarized mitochondria. The cell nuclei stained with DAPI. **(B)** Plot of the JC-1 fluorescence ratio (the ratio of red/green fluorescence of the dye in mitochondria).


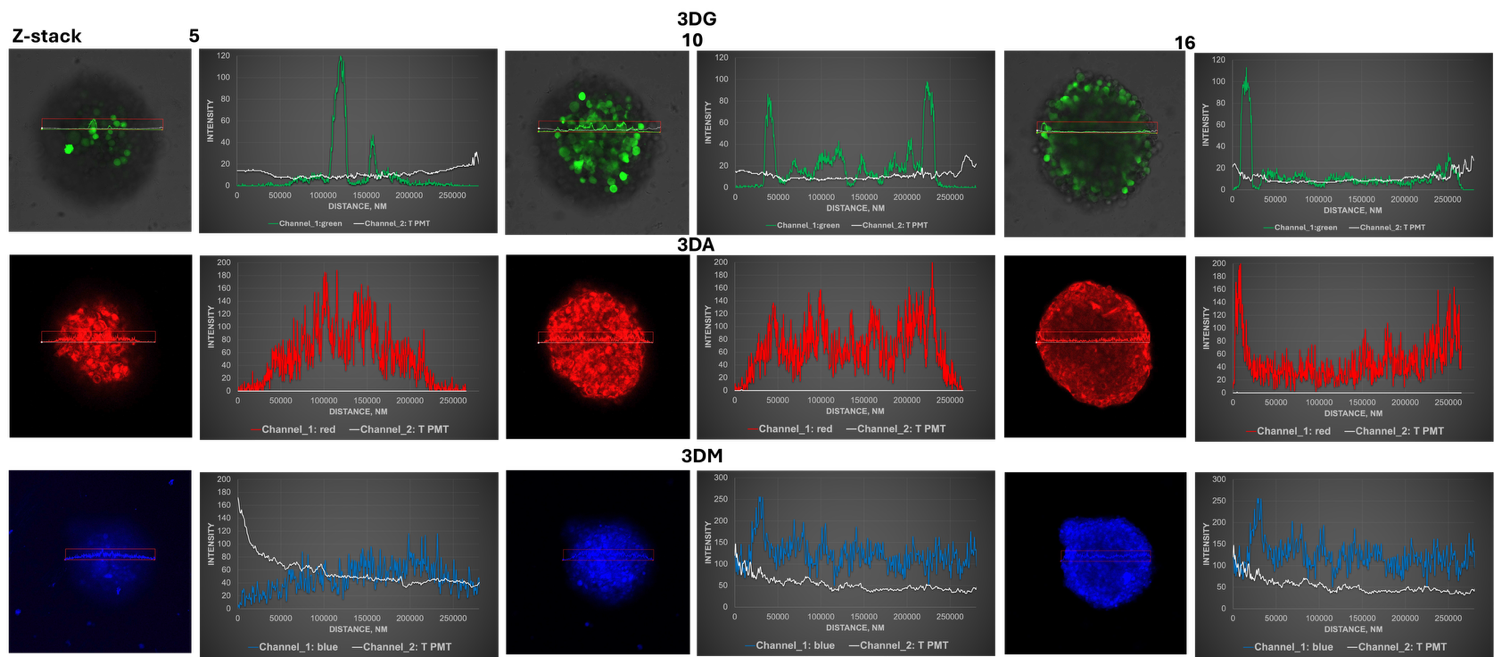


**Supplementary Figure S3. Intensity profiling along the center line of a homotypic spheroid evaluates 3D spatial organization and cellular density.** Confocal microscopy. Using 5, 10, and 16 Z-stacks provides varying resolutions that help track structural heterogeneity and define the core versus periphery of the spheroid.


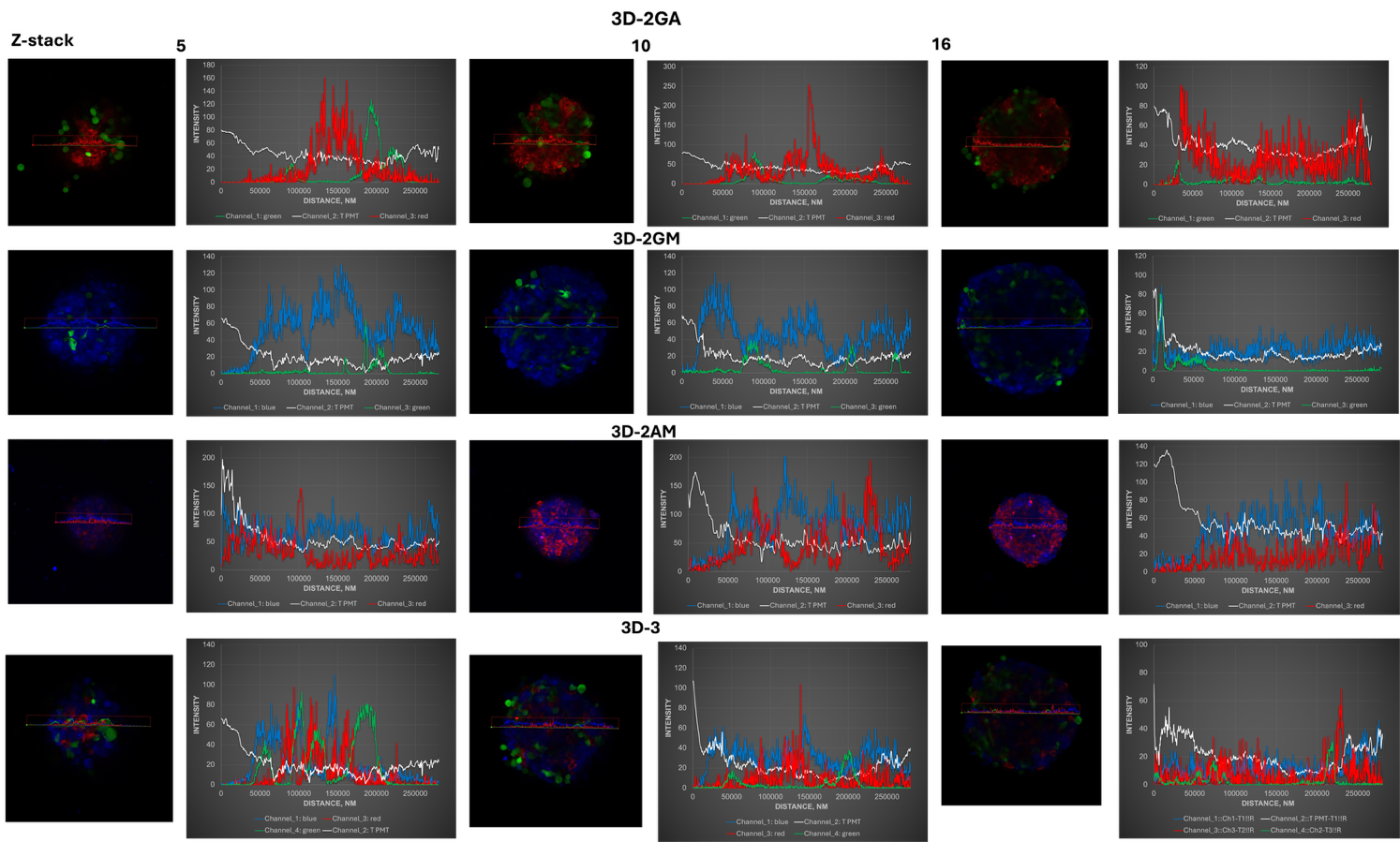


**Supplementary Figure S4. Intensity profiling along the center line of a heterotypic spheroid evaluates 3D spatial organization and cellular density.** Confocal microscopy. Using 5, 10, and 16 Z-stacks provides varying resolutions that help track structural heterogeneity and define the core versus periphery of the spheroid.

**3D-2AM 3D-2GA**


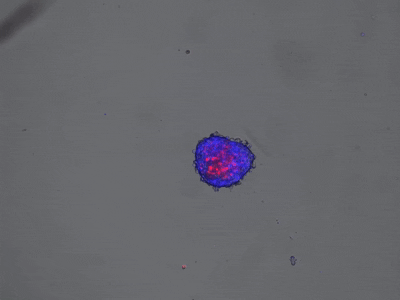

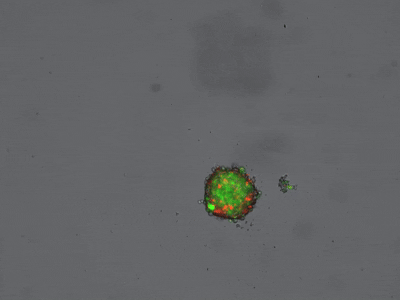


**3D-2GM 3D-3**


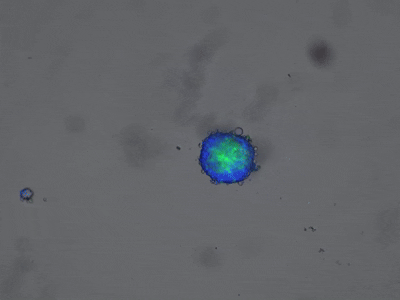

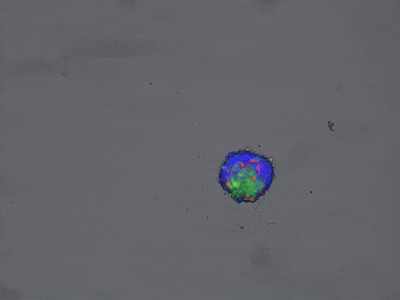


**Supplementary Figure S5.** **The video presents a visual documentation of cellular development and subsequent localization within a three-dimensional cultivation environment.**

**
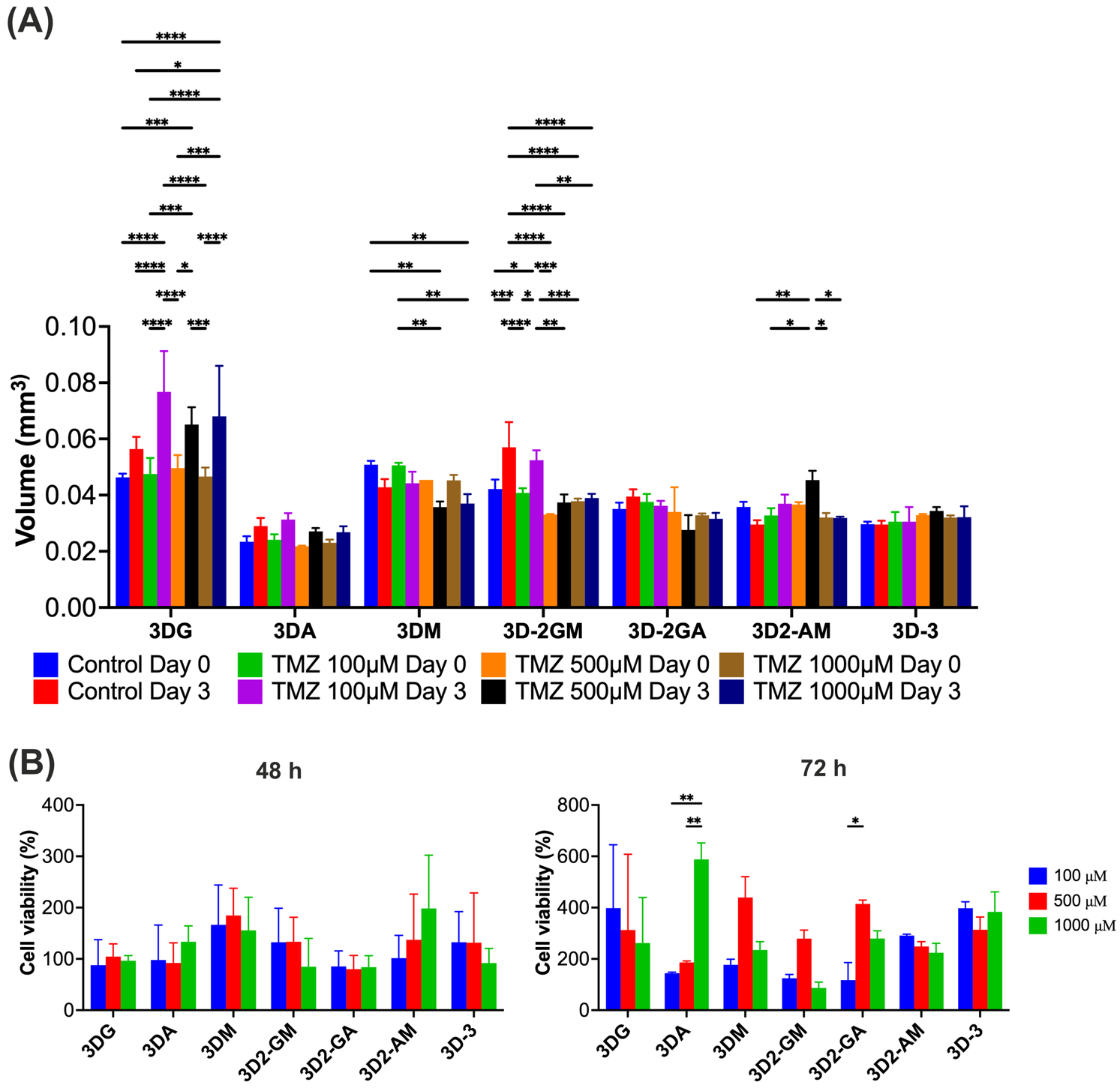
**

**Supplementary Figure S6. Response homo- and heterotypic spheroid to temozolomide (TMZ) treatment. (A)** Volume of the spheroid model during TMZ therapy. The first treatment was applied to spheroids on Day 0. The spheroids were treated with 100, 500 and 1000 μM of TMZ. **(B)** Cell viability determinations by MTT assay measuring absorbance at 570 nm for the control and different concentrations (TMZ) of sample. Data are presented as mean values (M) with standard deviation (SD), calculated from triplets of independent measurements. The difference between the experimental groups was statistically significant at *p <0.05; **p<0.01; *** p < 0.001; **** p < 0.0001 (one-way ANOVA with Shapiro-Wilk test).


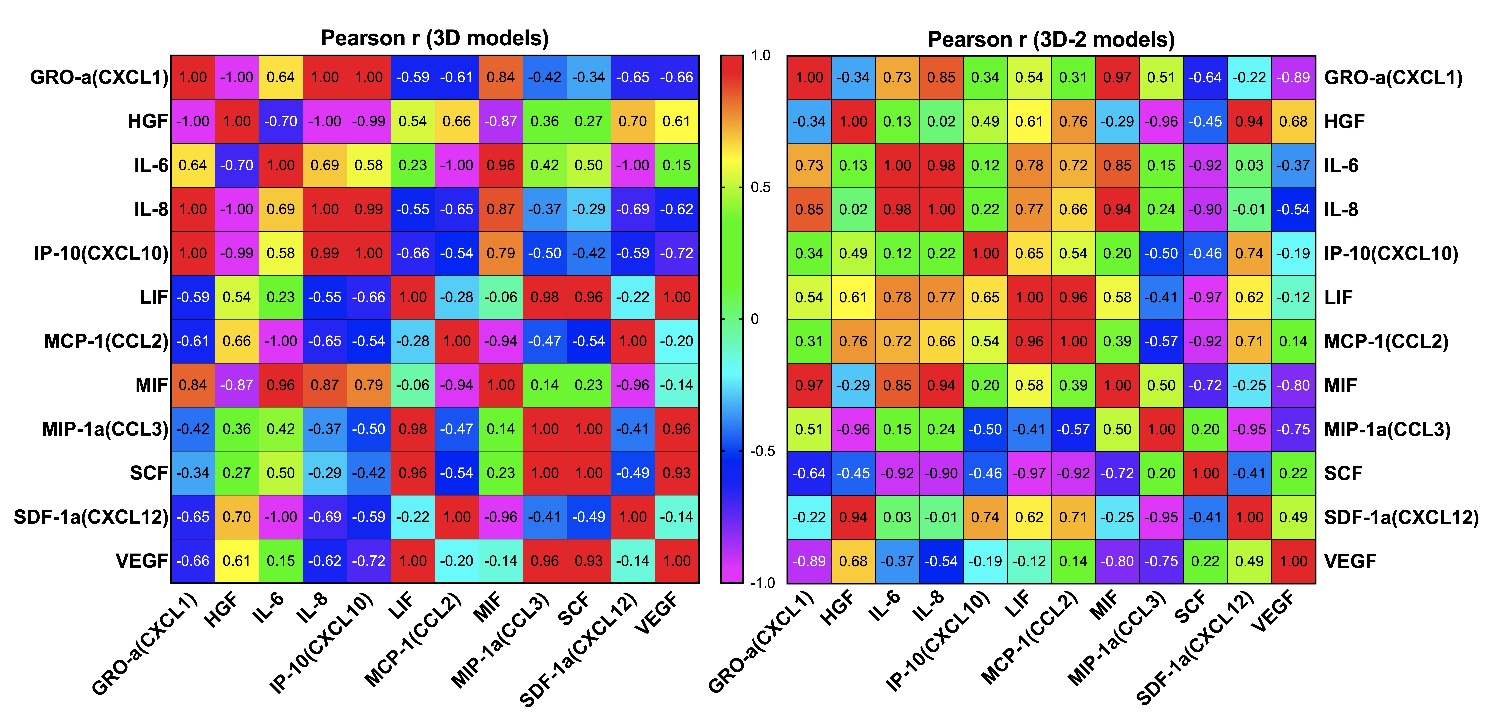


**Supplementary Figure S7.** **Correlation heatmap of cytokine concentrations**. Pearson linear correlation coefficients (r) are represented by a color scale: positive correlations (r > 0) are shown in shades of red, negative correlations (r < 0) in shades of blue, with color intensity proportional to the absolute value of the coefficient. Hierarchical clustering was performed using the complete linkage method with Euclidean distance. Data are presented as raw triplicate values of cytokine secretion without prior normalization or scaling. n = 3 for each variable.

**Correlation analysis of the cytokine network in homotypic and heterotypic 3D cultures**

In order to assess how cell-cell interactions reorganize cytokine regulatory networks, Pearson's correlation analysis was performed across two sets of experimental conditions: First, monotypic cultures (3DA, 3DG, 3DM) are defined as cells cultured in isolation, and second, heterotypic cultures (3D-2AM, 3D-2GM, 3D-2GA, 3D-3) are characterized as cells of different types in direct paracrine contact. The analysis revealed a dramatic reorganization of the cytokine network upon transition from isolated to co-culture, as clearly demonstrated by heat maps with hierarchical clustering (Figure S4).

In monotypic spheroids, the correlation matrix reveals the presence of two highly connected but functionally antagonistic clusters, reflecting the intrinsic regulatory programs inherent to each individual cell type. A nearly absolute positive correlation was identified between GRO-α (CXCL1), IL-8, and IL-6 (r ≥ 0.98), as well as with IP-10 (CXCL10) (r ≥ 0.99) and MIF (r ≥ 0.83). These findings suggest the formation of a proinflammatory/chemokine cluster. This tight co-expression suggests that, in the absence of intercellular interactions, the production of these cytokines is under the control of common intracellular signaling pathways, likely converging on the transcription factors NF-κB and AP-1.

The second cluster (neurotrophic/growth) is comprised of LIF, SCF, MIP-1α (CCL3), and VEGF, which exhibit a robust positive correlation with each other (r ≥ 0.93). A salient observation is the negative relationship between clusters, which is exemplified by the correlation between GRO-α and SCF (r = -0.34), the correlation between IL-6 and SCF (r = 0.50), and the negligible correlation between IL-6 and LIF (r = 0.23). This finding suggests the presence of two distinct regulatory programs in monotypic cultures, which are inherently incompatible and therefore obligate the cell to "choose" between them.

In the context of heterotypic cultures, a notable shift in correlation structure is observed, as evidenced by the reorganization of dendrograms on heat maps. This phenomenon indicates a qualitative change in the data representation, underscoring the importance of understanding the dynamics of these structures in the context of biological systems. Intercellular interactions have been demonstrated to "rewrite" intracellular regulatory programs, leading to the breakdown of some connections and the emergence of others. The disintegration of the proinflammatory cluster is characterized by a significant weakening of the tight association between GRO-α, IL-8, and IL-6, which is characteristic of monotypic cultures. Despite the persistent high correlation between IL-6 and IL-8 (r = 0.98), the association between IL-6 and GRO-α exhibits a decline, reaching r = 0.73, while the association between GRO-α and IL-8 experiences a decrease to r = 0.85. This finding suggests that within the microenvironment, the regulation of these cytokines becomes increasingly autonomous, presumably due to the presence of specific paracrine signals.

The replacement of old connections with new ones is indicative of the unique properties of the multicellular system. The strongest correlations in heterotypic cultures are observed between MCP-1 (CCL2) and LIF (r = 0.96), as well as between LIF and SCF (r = -0.97). These findings indicate a coupling of the regulation of monocyte recruitment and neurotrophic support in the microenvironment.

The "angiogenic-invasive module" is strengthened, with a strong positive correlation between HGF and SDF-1α (CXCL12) (r = 0.94) forming in heterotypic cultures, while this relationship was weaker in monotypic conditions (r = 0.70). This phenomenon may be indicative of the assembly of a singular signaling module that orchestrates the processes of stem cell invasion (CXCL12) and invasive potential (HGF) in response to the microenvironmental conditions. MIP-1α positioning in heterotypic MIP-1α (CCL3) cultures is characterized by a strong negative correlation with HGF (r = -0.96) and SDF-1α (r = -0.95), indicating its functional antagonism to the angiogenic-invasive module. This finding aligns with our earlier observation that MIP-1α production, which exhibits a marked increase in 3D-2GM, is repressed in the presence of astrocytes (3D-3). This observation potentially mirrors its function as a "danger signal" that is suppressed within a stable microenvironment.

The implementation of correlation analysis enabled the transition from a phenomenological description of cytokine levels to a systems-based understanding of the regulatory architecture of the glioma microenvironment. The obtained data demonstrate that the formation of a heterotypic microenvironment leads not only to quantitative changes in cytokine production, but also to a qualitative restructuring of the regulatory network, marked by the destruction of intracellular coregulatory modules and the emergence of new intercellular connections.


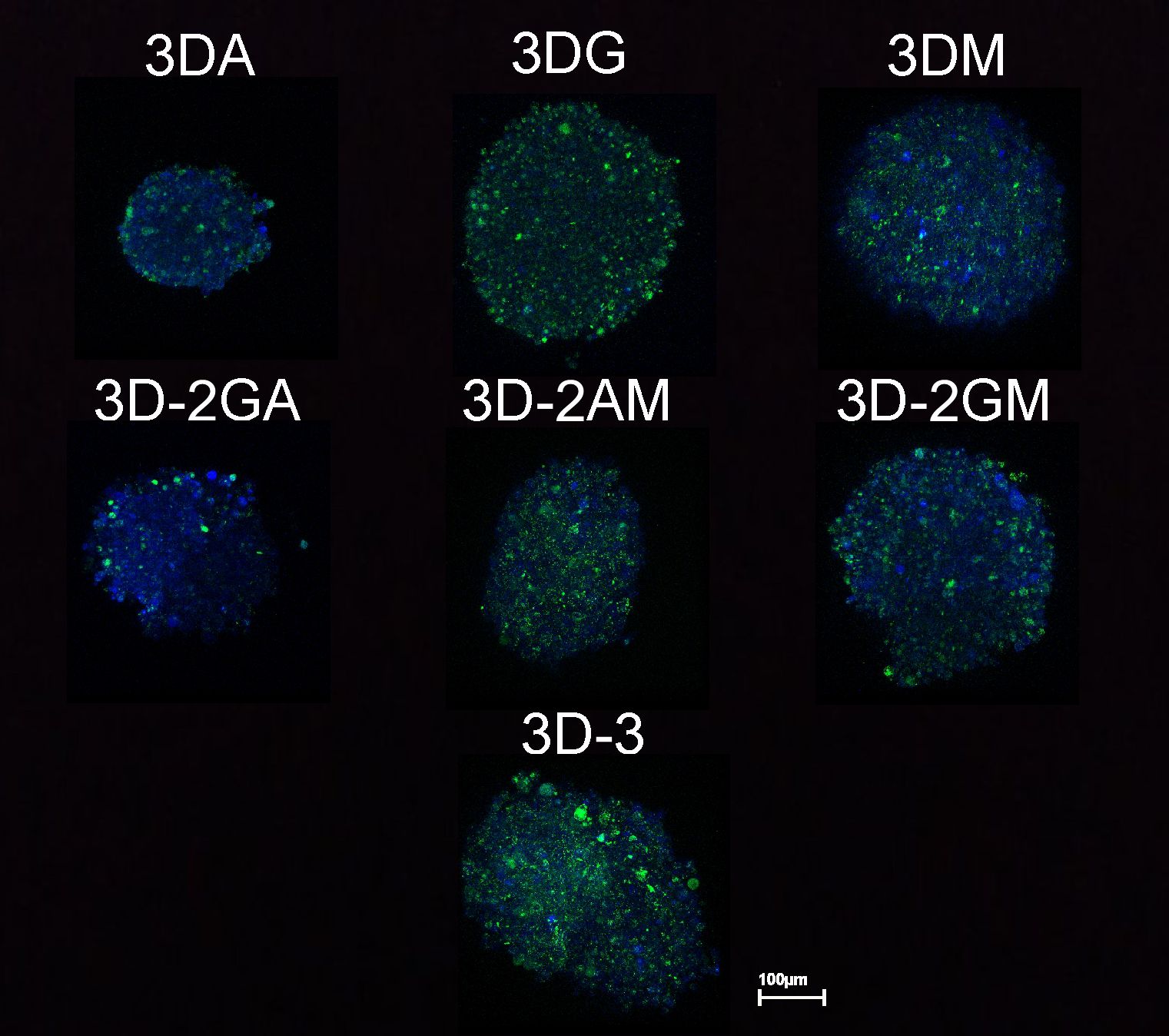


**Supplementary Figure S8.** **Analysis of barrier function in the spheroid model of brain tumors.** Penetration of FITC-dextran (70 kDa, green) into 3D spheroids. Nuclei were counterstained with Hoechst 33342 (blue). Confocal microscopy, scale bar: 100 µm


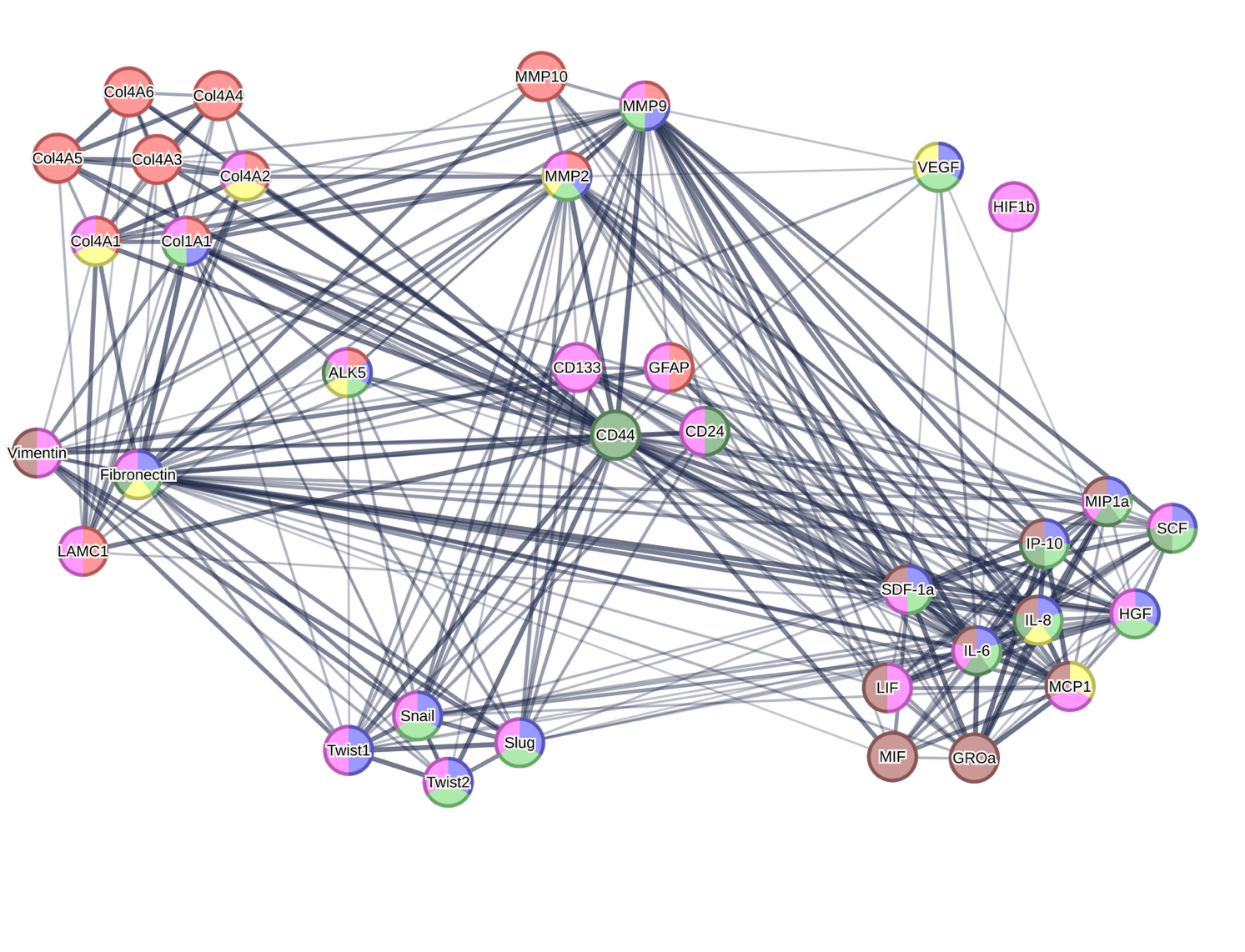


**Supplementary Figure S9.** **Protein-protein interaction network.** The network comprises 35 nodes and 270 edges (average node degree = 15.4, average local clustering coefficient = 0.809). The network has significantly more interactions than expected (expected number of edges = 46, PPI enrichment p value < 1.0e 16). This means that the proteins in the network exhibit many more connections among themselves than would be predicted for a random set of proteins of the same size and degree distribution drawn from the genome. Such a high level of enrichment indicates that these proteins are at least partially biologically connected as a functional group, reflecting genuine biological relationships rather than random chance.

The molecular characterization of the models yielded several noteworthy results. A subsequent summary network of protein-protein interactions was constructed and determined using the STRING database (Search Tool for the Retrieval of Interacting Genes/Proteins) (Szklarczyk et al., 2025). This database combines information on predicted and experimentally confirmed interactions between proteins, thereby demonstrating a high degree of functional connectivity between expressed and secreted molecules in the immunosuppressive TME of glioma (Figure S6). The identified protein-protein interaction network encompassed a total of 35 targets. Network nodes are color-coded according to the Gene Ontology (GO) biological processes in which these proteins are predominantly involved. The analysis identifies several functional clusters reflecting key aspects of tumor physiology, such as extracellular matrix remodeling, regulation of cell motility, angiogenesis, differentiation, and immune response.

The red cluster corresponds to the following Gene Ontology (GO) term: extracellular matrix organization (GO:0030198). The upper left region contains a dense cluster of proteins associated with the formation and remodeling of the extracellular matrix (ECM). This cluster includes collagens (Col4A1, Col4A2, Col4A3, Col4A4, Col4A5, Col4A6, Col1A1), fibronectin, laminin (LAMC1), and metalloproteinases (MMP2, MMP9, MMP10). These proteins are critical for maintaining the structural integrity of the tissue and facilitating cell migration. The interaction between type I collagens and MMP2 and MMP9 is particularly notable, indicating active proteolysis of basement membranes - a process essential for cell invasion.

The presence of blue and light green clusters indicates a positive regulation of cell motility and migration, as indicated by the Gene Ontology (GO) terms "GO:2000147" and "GO:0030335." These two processes exhibit a high degree of similarity with respect to their protein sets. These groups include chemokines and growth factors (e.g., SDF-1α, MIP-1α, SCF, IP-10, IL-6, IL-8), as well as epithelial-mesenchymal transition transcription factors (e.g., SNAIL, SLUG, TWIST1, TWIST 2). Proteins in this module, which are colored blue and light green, have been shown to interact with ECM components. For example, HGF has been observed to stimulate MMP expression, and motility-associated CD44 has been demonstrated to directly interact with fibronectin and collagens. Consequently, this segment of the network facilitates the integration of signals that regulate cell movement.

The yellow cluster corresponds to the term "angiogenesis" (GO:0001525). The pivotal elements in this context are vascular endothelial growth factor (VEGF), interleukin-8 (IL-8), and the aforementioned metalloproteinases, matrix metalloproteinase 2 (MMP2) and matrix metalloproteinase 9 (MMP9). VEGF and HIF-1β are combined into a central regulatory hub that controls the formation of new vessels. It is noteworthy that VEGF is associated with MMP2 and MMP9, which facilitate the release of growth factors from the matrix. Furthermore, IL-8 (CXCL8) and HGF have been shown to function as pro-angiogenic signals by binding to receptors on endothelial cells. Despite the fact that only eight proteins are formally included in this cluster, their high degree of connectivity within the network indicates a key role for angiogenesis in the overall context of tumor activity.

The dark green cluster indicates cell activation (GO:0001775). This cluster includes proinflammatory cytokines and chemokines (IL-6, IL-8, MIP-1α, SCF), as well as CD44. In this context, cell activation is comprehended in a broad sense, encompassing a range of processes including the induction of proliferation and migration. Of particular interest is the overlap between activation proteins and angiogenesis factors, such as IL-6 and VEGF, which reflects the established connection between inflammation and vascular growth.

The purple cluster corresponds to cell differentiation (GO:0030154). This is the largest group (24 proteins) and is not isolated, but rather permeates the entire network. The presence of specific differentiation markers (CD133, CD24, GFAP, ALK5) has been observed to be associated with the transcription factors (SNAIL, SLUG, TWIST1/2) and signaling molecules (HGF, LIF, SCF). CD133, a stem cell marker, has been observed to interact with CD44 and integrins, thereby connecting differentiation to migration and adhesion processes. LIF (leukemia inhibitory factor) has also been demonstrated to link this module to differentiation processes.

The brown cluster corresponds to the category of immune response (GO:0006955). The most compact group, which contains 10 proteins, includes chemokines and cytokines, such as MIP1a, MCP-1, SDF-1α, IL-6, IL-8, LIF, MIF, and GRO-α. These proteins are secreted during inflammation and act as chemoattractants for leukocytes. Notably, these cells establish intimate associations with metalloproteinases and growth factors, thereby elucidating the manner in which immune signals modulate the motility of stromal and tumor cells.

A comprehensive analysis of the network reveals the presence of multiple hubs exhibiting high levels of interconnectedness (SDF-1α, MMP9, VEGF, IL-8, SNAIL), which serve as conduits, connecting disparate color clusters. For instance, MMP9 (red, blue, light green, purple) is involved in collagen degradation, migration stimulation, and angiogenesis. Twist1 (blue, purple) has been shown to regulate the expression of numerous ECM proteins and metalloproteinases, thereby establishing a link between EMT, motility, and matrix remodeling. This multifunctionality mirrors the biological reality in which the same proteins can perform different roles depending on the context.
